# Oxidative Potential Versus Biological Effects: A Review on the Relevance of Cell-Free/Abiotic Assays as Predictors of Toxicity from Airborne Particulate Matter

**DOI:** 10.3390/ijms20194772

**Published:** 2019-09-26

**Authors:** Johan Øvrevik

**Affiliations:** 1 Department of Air Pollution and Noise, Division of Environmental Medicine, Norwegian Institute of Public Health, 0213 Oslo, Norway; johan.ovrevik@fhi.no; Tel.: +47-21-07-64-08; 2 Department of Biosciences, Faculty of Mathematics and Natural Sciences, University of Oslo, 0316 Oslo, Norway

**Keywords:** air pollution, particulate matter, oxidative stress, health effects, inflammation, cellular responses, mechanisms of effects

## Abstract

*Background and Objectives*: The oxidative potential (OP) of particulate matter (PM) in cell-free/abiotic systems have been suggested as a possible measure of their biological reactivity and a relevant exposure metric for ambient air PM in epidemiological studies. The present review examined whether the OP of particles correlate with their biological effects, to determine the relevance of these cell-free assays as predictors of particle toxicity. *Methods*: PubMed, Google Scholar and Web of Science databases were searched to identify relevant studies published up to May 2019. The main inclusion criteria used for the selection of studies were that they should contain (1) multiple PM types or samples, (2) assessment of oxidative potential in cell-free systems and (3) assessment of biological effects in cells, animals or humans. *Results*: In total, 50 independent studies were identified assessing both OP and biological effects of ambient air PM or combustion particles such as diesel exhaust and wood smoke particles: 32 in vitro or in vivo studies exploring effects in cells or animals, and 18 clinical or epidemiological studies exploring effects in humans. Of these, 29 studies assessed the association between OP and biological effects by statistical analysis: 10 studies reported that at least one OP measure was statistically significantly associated with all endpoints examined, 12 studies reported that at least one OP measure was significantly associated with at least one effect outcome, while seven studies reported no significant correlation/association between any OP measures and any biological effects. The overall assessment revealed considerable variability in reported association between individual OP assays and specific outcomes, but evidence of positive association between intracellular ROS, oxidative damage and antioxidant response in vitro, and between OP assessed by the dithiothreitol (DDT) assay and asthma/wheeze in humans. There was little support for consistent association between OP and any other outcome assessed, either due to repeated lack of statistical association, variability in reported findings or limited numbers of available studies. *Conclusions*: Current assays for OP in cell-free/abiotic systems appear to have limited value in predicting PM toxicity. Clarifying the underlying causes may be important for further advancement in the field.

## 1. Introduction

Airborne particulate matter (PM) represents one of the major environmental risk factors for disease and premature death worldwide, and has been associated with development or exacerbation of a number of adverse health effects including asthma, chronic obstructive pulmonary disease (COPD), cancer, cardiovascular disease (CVD), metabolic and neurological disorders and adverse birth effects [1,2,3,4,5,6]. Activation of inflammatory reactions is considered a central driving mechanism for many adverse health effects from particle exposure [3,7,8,9]. In addition, particle exposure may affect cell proliferation, induce cell-cycle alterations, damage DNA and other macromolecules and cause different forms of cell death [10,11,12,13].

The exact mechanisms through which PM trigger these biological effects in exposed cells or organisms, remains to be determined. PM is highly complex and may carry an abundancy of well-known toxins including transition metals, polycyclic aromatic hydrocarbons (PAHs), quinones and bacterial endotoxins, which are considered potential mediators of PM-toxicity [9]. However, a dominating theory is that particles trigger cellular effects through the formation of reactive oxygen species (ROS) or oxidation of biomolecules. The oxidative stress paradigm in particle toxicology encompasses both primary oxidative effects from the particles or adhered particle components, as well as secondary ROS production in exposed cells or tissues. In the strictest form, the oxidative stress paradigm postulates that the biological reactivity of a particle is due to its oxidative potential (OP): The ability to produce ROS or oxidize target substrates directly in contact with biological fluids or cellular molecules. Although non-oxidant mediated mechanisms of PM-toxicity are well known [9], the concept of OP as determinant for PM-toxicity has received considerable attention and it has been linked to all fields of particle toxicology and all the main outcomes of particle exposure, as extensively reviewed by others [14,15,16,17,18,19].

The OP of particles can be measured in cell-free/abiotic systems, and has been suggested as a promising screening method to predict the biological reactivity of particles [20,21]. Acellular OP has also been suggested as a toxicological relevant feature of ambient air PM that could provide improved exposure metrics in epidemiological studies, compared to more conventional particle-metrics such as mass, surface area or number concentrations [22,23,24]. However, the concept of OP as a triggering mechanism for particle toxicity lacks clear, precise definitions. While terms such as oxidative potential or oxidative capacity are most frequently used to describe the particles inherent (primary) abilities to produce ROS or oxidize target substrates directly in contact with biological fluids or cellular molecules, some also incorporate the ability to induce intracellular ROS levels and oxidative stress responses in exposed cells or tissues. There is a significant difference between these two events. While the former represents a potential triggering mechanism of effects, the later often arise from stimulating endogenous production of ROS or RNS (reactive nitrogen species) and may therefore rather be seen as an effect of the exposure [9]. To avoid further confusion, the term “oxidative potential” or “OP” will in this paper strictly be used to describe the inherent abilities of particles or particle-bound components to produce ROS or oxidize substrates, thus measurable under abiotic/cell-free conditions.

To further complicate the scenario, the term ROS also lack precision as it covers a variety of reactive species including hydrogen peroxide (H_2_O_2_), superoxide (O_2_^●−^), singlet oxygen (^1^O_2_), hydroxyl radicals (^●^OH), ozone (O_3_), hypohalous acids (HOX) and organic peroxides. These different species varies considerably in reactivity and longevity, and may elicit different effects in cells and tissues. As pointed out elsewhere [25,26], they are also difficult to distinguish and quantify due to low specificity of available assays (Table 1). Many organic compounds such as PAHs are redox-active only after metabolic activation by cellular enzymes, and are thus not detected by current acellular OP assays. Finally, it should also be considered that establishing a clear causal link between OP in abiotic systems and biological effects is inherently difficult, as antioxidants does not clearly distinguish between the role of primary oxidative events induced by the particles, and the secondary endogenous redox responses of exposed cells [9]. Due to these uncertainties and potential caveats, the precise role of OP in particle toxicity should be interpreted with some caution. The purpose of this paper was therefore to review to what extent OP in cell-free/abiotic systems provides a relevant measure of PM-toxicity, correlating with the biological effects observed in PM-exposed cells, animals and humans.

## 2. Literature Search and Selection of Studies

PubMed was searched for publications containing the terms “particles” or “particulate matter” in combination with the terms “oxidative” or “reactive oxygen” in the title or abstract published up to April 2019. The search “particles[Title/Abstract] AND oxidative[Title/Abstract]” alone, retrieved 3606 publications. The search results were then reduced by adding terms such as “oxidative potential”, “acellular”, “air pollution”, “PM”, “combustion particles”, “diesel exhaust”, “DEP”, “wood smoke”, etc., or common assays for detection of OP (“ESR”, “EPR”, “DTT”, etc.). However, due to inconsistent use of terminology and the massive amount of literature on particles and oxidative stress, discriminating between studies assessing acellular OP and cellular redox responses, systematic search strategies proved insufficient to identify relevant publications. Therefore, the reference lists of central reviews on particle toxicity and oxidative stress were also checked for relevant papers, and the Web of Science and Google Scholar databases was used to search the citation network (cited references and citing articles) of identified studies for other relevant publications.

The main inclusion criteria used for the selection of studies were that they should contain:

(1) Multiple particles types or samples;
(2) Assessment of OP in cell-free systems;
(3) Assessment of biological effects in cells, animals or humans.

Studies that did not meet all three criteria were omitted. Furthermore, if two or more studies were based on identical particle samples and utilized measures of OP obtained from the same analysis, these were not considered as independent, and therefore grouped and counted as one study.

In total 58 studies were identified exploring both the OP and biological effects of PM and/or combustion particles in cells, animals or humans [27,28,29,30,31,32,33,34,35,36,37,38,39,40,41,42,43,44,45,46,47,48,49,50,51,52,53,54,55,56,57,58,59,60,61,62,63,64,65,66,67,68,69,70,71,72,73,74,75,76,77,78,79,80,81,82,83,84]. Among these were three cases of multiple studies based on the same particle samples and measurements of OP: Two studies on in vitro and in vivo effects of a set of wood smoke and PM samples [45,46], and six studies from a series of PM exposures of human volunteers under the RAPTES (Risk of Airborne Particles: A Toxicological–Epidemiological Hybrid Study) project [67,68,69,70,71,72], and three epidemiological studies by Weichenthal and colleagues [80,81,82]. These were grouped and counted as only three independent studies. Notably, three additional studies from one research group assessing association between modeled OP and hospital visits for respiratory and cardiovascular disease could possibly also have been grouped, but were analyzed separately due to some variability in samples used for OP measurements and modeling [66,75,76]. Furthermore, as this review has assessed experimental studies in cells/animals and human studies separately, one study performing both experimental studies in vitro and epidemiological assessment in humans was counted twice [49]. Thus, in total 50 independent studies were identified (Tables S1 and S2, Supplementary materials): 32 in vitro or in vivo studies exploring effects in cells or animals, and 18 clinical or epidemiological studies exploring effects in humans. Furthermore, 31 studies assessed the association between OP and biological effects by statistical analysis. However, two studies obtained statistically significant associations only after assessing different PM samples separately, and were therefore not considered to show an overall statistically significant association [38,53]. The OP assays most commonly used in the identified studies were electron spin resonance (ESR) with 5,5-dimethyl-pyrroline N-oxide (DMPO) as spin trap, the dithiothreitol (DTT) assay, as well as ascorbic aid (AA) and glutathione (GSH) depletion. Other OP assays (Table 1) were used more sporadically and rarely in studies assessing the association between OP and biological effects by statistical analysis (Tables S1 and S2, supplementary materials).

In cases where the relationship between OP and biological effects were compared by statistical analysis (such as linear regression), the studies were marked with either “statistical significant correlation/association” or “no statistical significant correlation/association” (Tables S1 and S2, supplementary materials). In cases where no statistical analysis was available, the studies were marked with either “possible association” or “no apparent association” based on a comparison of the rank order of OP and rank order of potency to induce biological effects induced by the different particulates tested in the study (Tables S1 and S2, supplementary materials). Importantly, the main conclusions of this review have been based on the 29 studies were correlation between OP and biological effects was assessed by statistical analysis [29,32,43,44,49,50,52,55,56,57,58,59,60,63,64,65,66,67,68,69,70,71,72,73,74,75,76,77,78,79,80,81,82,83,84].

Due to the mixed search strategy applied, this work should not be considered a systematic review. However, the material presented and discussed represents, by far, the most comprehensive overview of studies on OP and biological effects presented to date. A detailed overview of all the 58 identified publications, including PM types/samples, OP assay, concentrations used for OP assays and exposure concentrations, biological endpoints assessed and summary of key findings (association/no association), is given in the two tables in Supplementary Materials (Tables S1 and S2). All figures presented below are based on the information available in these two tables.

## 3. Results

### Consistency of Associations Between OP and Biological Effects

**Figure 1:**
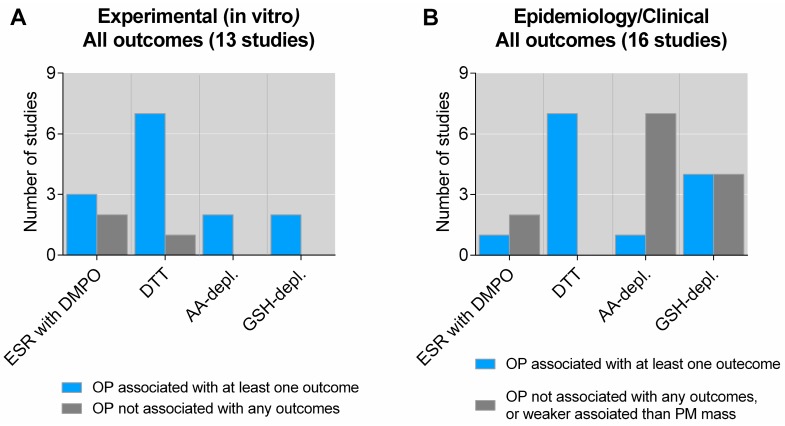
Overview of studies exploring the association between OP and biological effects of particulate matter (PM). The figure displays the number of experimental studies in vitro (**A**) and epidemiological/clinical studies in humans (**B**) exploring the association between specific OP assays and biological effects by statistical analyses. As some studies have explored OP by several different assays, the sum of the individual columns exceeds the total number of studies given in the figure title. Details on the individual studies are given in Tables S1 and S2 (supplementary materials). AA depl.—ascorbic acid depletion; DMPO—5,5-dimethyl-pyrroline N-oxide; DTT—dithiothreitol; ESR—electron spin resonance; GSH depl.—reduced glutathione depletion; OP—oxidative potential.

Statistical significant associations between OP and biological effects were reported in 22 of the 29 independent studies were statistical analysis were applied [29,32,43,44,49,52,57,58,59,60,63,65,66,74,75,76,77,78,79,80,81,82,83,84]: 10 independent studies reported that at least one OP measure was statistically significantly associated with all endpoints examined [43,49,52,57,63,66,78,79,80,81,83], while additional 12 independent studies reported that at least one OP measure was significantly associated with at least one effect outcome [29,32,44,58,59,60,65,74,75,76,77,82,84]. By contrast, seven studies reported no significant correlation/association between any OP measures and any biological effects [50,55,56,64,65,67,68,69,70,71,72,73] (Figure 1, and Tables S1 and S2, supplementary materials). As some publications were grouped due to shared PM samples and OP analysis, the total number of publications referenced above, exceeds the number of studies considered as independent. Notably, among the positive associations reported, one study reported statistically significant associations between OP and effects only after excluding a selected sample from the analysis [29], one reported very weak associations, which were increased after excluding a subset of particles for which the OP did not correlate with biological effects [43], and one appeared to be based on unrealistically high in vitro concentrations [52]. These three studies were nevertheless included as positive associations in the analysis below.

Of the 22 studies that reported a statistically significant association between at least one OP measure and at least one or more biological effects, were 10 experimental in vitro or in vivo studies in cells or animals [29,32,43,44,49,50,52,55,56,57,58,59,60], and 11 epidemiological or clinical studies in humans [49,63,64,65,66,67,68,69,70,71,72,73,74,75,76,77,78,79,80,81,82,83,84] (Figure 1). Of the seven independent studies reporting no statistical significant association between any OP measure and any biological effects were three independent experimental in vitro or in vivo studies [50,55,56], and four independent epidemiological or clinical studies [64,65,67,68,69,70,71,72,73] (Figure 1). At glance, this may give the impression of a marked overweight of positive associations between OP and biological effects. It seems likely that this at least in part could be the reason for the general assumption often encountered in the literature, that OP may provide a good prediction PM toxicity. However, an alternative way to present this is that 19 of the 29 studies (65%) showed that OP was not statistically significantly associated with at least one or more biological effects examined [29,32,44,50,55,56,58,59,60,64,65,67,68,69,70,71,72,73,74,75,76,77,82,84].

**Figure 2:**
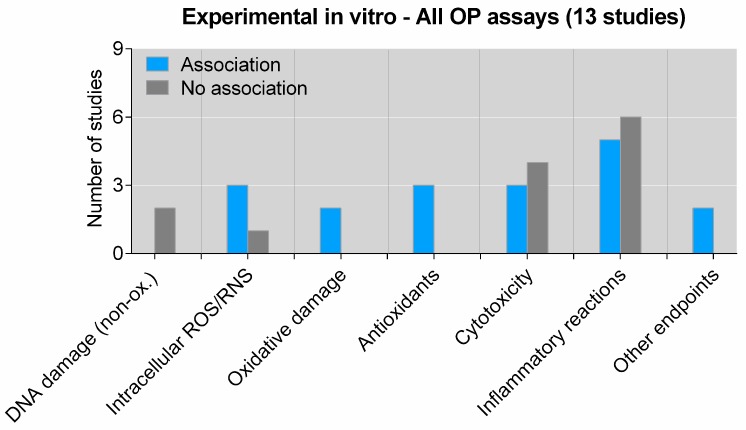
Association between all OP assays (pooled) and biological effects of PM in experimental studies in vitro. The figure displays the number of studies showing an association or no association between OP measured by any assay and specific biological effects in vitro (cell cultures). “Oxidative damage” includes lipid peroxidation and oxidative DNA damage. “Other endpoints” include cellular signaling and proliferation. As some studies have explored association between OP and several different biological effects, the sum of the individual columns exceeds the total number of studies given in the figure title. Details on the individual studies are given in Tables S1 and S2 (supplementary materials). Non-ox—non-oxidative; OP—oxidative potential; ROS—reactive oxygen species; RNS—reactive nitrogen species.

**Figure 3:**
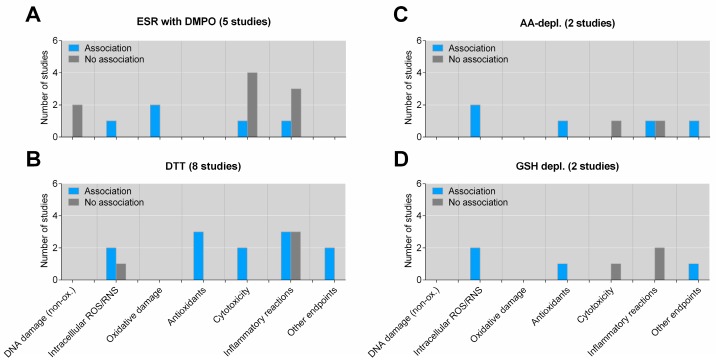
Association between specific OP assays and biological effects of PM in experimental studies in vitro. The figure displays the number of studies showing an association or no association between OP measured by electron spin resonance (ESR) with DMPO as a spin trap (**A**), DTT assay (**B**), ascorbic acid (AA)-depletion (**C**) or reduced glutathione (GSH)-depletion (**D**)and specific biological effects in vitro (cell cultures). “Oxidative damage” includes lipid peroxidation and oxidative DNA damage. “Other endpoints” include cellular signaling and proliferation. As some studies have explored association between OP and several different biological effects, the sum of the individual columns exceeds the total number of studies given in the figure title. Details on the individual studies are given in Tables S1 and S2 (supplementary materials). AA-depl.—ascorbic acid depletion; DMPO—5,5-dimethyl-pyrroline N-oxide; DTT—dithiothreitol; ESR—electron spin resonance; GSH depl.—reduced glutathione depletion.

The identified studies utilized a number of different OP measures and biological endpoints, and most investigated effects on multiple biological effects. To further analyze the data, biological endpoints were grouped in various categories. For experimental studies, endpoints were grouped into DNA damage (non-oxidative), ROS/RNS production (measured in cells or animals), oxidative damage (to DNA, proteins and lipids), antioxidant responses, cytotoxicity, inflammatory reactions and “other endpoints” (CYP1A1-expression and phagocytosis). Results obtained by in vitro and in vivo studies were separated. When analyzing reported statistical associations with these specific outcome groups for all OP assays (pooled), it becomes clear that a greater number of reported positive associations with OP were restricted to associations with intracellular ROS formation, oxidative damage and antioxidant responses in vitro (Figure 2). The number of available studies assessing the association between OP and these three individual outcomes was also relatively low (*n* ≤ 3). No study has reported a statistically significant association between OP and non-oxidative DNA damage (strand breaks, adduct formation, etc.), but two studies found that DNA damage was not associated with OP. For the remaining two specific categories, cytotoxicity and inflammation, there seems to be a slight predominance of negative studies, suggesting no statistically significant association with OP (Figure 2). Assessing the reported associations for specific OP assays and these specific outcome groups further strengthened the notion that reproducible statistical significant associations with OP were limited to the redox-related outcomes (Figure 3, and Tables S1 and S2, supplementary materials). The only exception was the DTT assay, which also appeared to be positively associated with in vitro cytotoxicity, but only two studies were available for this outcome (Figure 3B). In general, few studies (*n* = 1–3) were available for most combinations of specific OP assays and specific effect outcomes, hampering strong conclusions.

Additional 20 experimental in vitro and in vivo studies assessed OP and biological effects without analyzing the association by statistical methods [27,28,30,31,33,34,35,36,37,38,39,40,41,42,45,46,47,48,51,53,54]. These were also evaluated by comparing the rank order of potency for OP and biological effects induced by the particles. If the rank order of OP and ability to induce a biological effect were comparable, the study was denoted as “possible association”. If the rank order clearly differed the study was denoted “no apparent association” (Tables S1 and S2, supplementary materials). When adding these 20 identified experimental studies where statistically significant associations were not included to the analysis, a marked predominance of studies suggesting no association between OP and all specific outcome groups can be observed, with the exception of in vitro increases in antioxidant levels (Figure S1, Supplementary Materials). This is also the case when separating this material according to the specific OP assays used (Figure S2, Supplementary Materials). However, the relevance of this should be interpreted with care, as such evaluations may be more prone to subjective interpretations. There is another obvious caveat of the approach: If the rank order of potency differs only for one or a few among many PM samples (i.e., outliers), there could still be an overall statistical significant association between OP and effects. On the other hand, if a statistical significant correlation between OP and a biological effect is present, despite marked differences in rank order of OP and effects, one could also argue that OP not sufficiently accounts for the ability to induce the biological effect in question. Nevertheless, due to the possibility of misinterpretation of these data, the main conclusions of this review was based on the studies were statistical analysis was employed by the authors.

**Figure 4:**
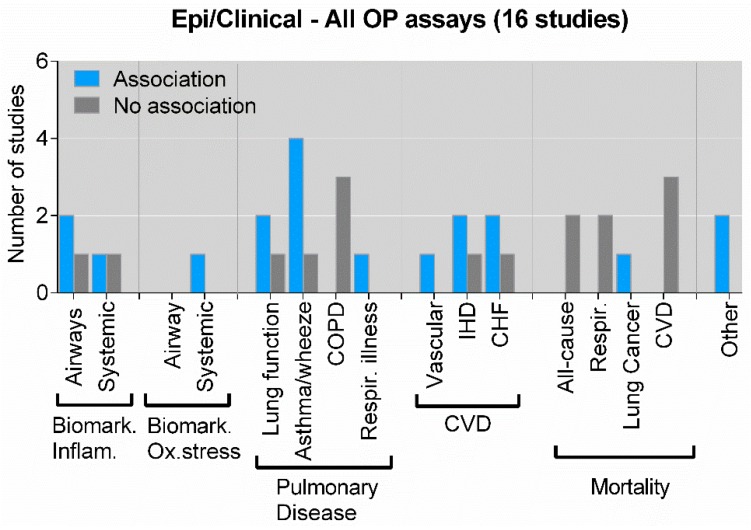
Association between all OP assays (pooled) and biological effects of PM in epidemiological and clinical studies. The figure displays the number of studies showing an association or no association between OP measured by any assay and specific biological effects in humans assessed by epidemiological or clinical studies. “Other endpoints” include pre-term birth and diabetes. As some studies have explored the association between OP and several different biological effects, the sum of the individual columns exceeds the total number of studies given in the figure title. Details on the individual studies are given in Tables S1 and S2 (supplementary materials). Biomark. Inflam.—biomarkers of inflammation; Biomark. Ox.stress—biomarkers of oxidative stress; COPD—chronic obstructive pulmonary disease; CVD—cardiovascular disease; CHF—coronary heart failure; IHD—ischemic heart disease; Respir.—respiratory.

Endpoints assessed in epidemiological and clinical studies were also grouped, but in other categories: Biomarkers of inflammation (airways and systemic), biomarkers of oxidative stress (airways and systemic), pulmonary disease (lung function, asthma/wheeze, chronic obstructive pulmonary disease (COPD) and respiratory illness), CVD (vascular disease, ischemic heart disease (IHD) and coronary heart failure (CHF)), mortality (all cause, respiratory, lung cancer and CVD) and other outcomes (pre-term birth and diabetes). When assessing the reported statistical associations between all OP assays (pooled) and specific outcome groups, there seem to be a marked predominance of reported positive associations between OP and asthma/wheeze, and a slight predominance of positive associations between OP and airway inflammation, lung function and CVD (Figure 4). By contrast, there was a marked predominance of negative associations reported for OP and COPD as well as mortality (Figure 4).

**Figure 5:**
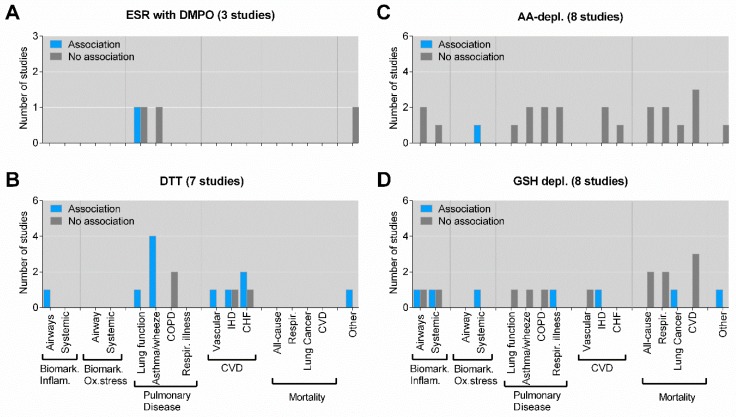
Association between specific OP assays and biological effects of PM in epidemiological and clinical studies. The figure displays the number of studies showing an association or no association between OP measured by ESR with DMPO as spin trap (**A**), DTT assay (**B**), AA-depletion (**C**) or GSH-depletion (**D**), and specific biological effects in humans assessed by epidemiological or clinical studies. “Other endpoints” include pre-term birth and diabetes. As some studies have explored association between OP and several different biological effects, the sum of the individual columns exceeds the total number of studies given in the figure title. Details on the individual studies are given in Tables S1 and S2 (supplementary materials). AA—ascorbic acid; Biomark. Inflam.—biomarkers of inflammation; Biomark. Ox.stress—biomarkers of oxidative stress; COPD—chronic obstructive pulmonary disease; CVD—cardiovascular disease; CHF—coronary heart failure; DMPO—5,5-dimethyl-pyrroline N-oxide; DTT—dithiothreitol; ESR—electron spin resonance; GSH—reduced glutathione; IHD—ischemic heart disease; Respir.—respiratory.

Further dividing the epidemiological and clinical studies by specific OP assays revealed some interesting information on the different assays used. ESR with DMPO was only used in three identified studies, and seems at present to provide limited support for any clear association with effects (Figure 5A). The DTT assay, on the other hand, has been used in seven studies and shows a clear association with asthma/wheeze (four positive and no negative studies). However, three out of the four positive associations between OP and asthma/wheeze were reported by the same research group, using modeled OP (DTT) for the overlapping region and time period [66,75,76], and may therefore not be considered as truly independent observations. Positive associations between DTT and other endpoints including airway inflammation, lung function, vascular disease and “other outcomes” (diabetes) has also been reported, but only one study was available for each endpoint, hampering strong conclusions. For IHD and CHF mixed associations have been reported, but also here the number of studies are limited (Figure 5B). By contrast, studies using the AA-depletion assay have almost consistently failed to find any positive associations between this OP assay and any effect outcomes, in epidemiological and clinical studies (Figure 5C). The GSH-depletion assay shows somewhat more mixed associations, and few available studies for each outcome (*n* = 1–2) hampers any conclusions regarding its potential association with effects in humans (Figure 5D).

## 4. Discussion

Assessment of the identified studies revealed a considerable variability in reported association between individual OP assays and specific outcomes. There seem to be a predominance of positive associations reported for OP and redox-related responses in in vitro cell models, including intracellular ROS generation, oxidative damage to macromolecules and antioxidant response. By contrast, a predominance of studies reported that in vitro cytotoxicity, inflammatory reactions and non-oxidative DNA-damage is not associated with the OP of PM. No experimental animal studies exploring the statistical association between OP and effects were identified. Furthermore, epidemiological and clinical studies in humans suggest a positive association between OP measured by the DDT assay and asthma/wheeze in epidemiological and clinical studies. For all other outcomes assessed, the number studies on the association between a specific OP assay and a specific endpoint was either too low to conclude, or conflicting results were reported from different studies (Figure 3 and Figure 5). Notably the AA-depletion assay has almost completely failed to be associated with effects in all studies on humans, suggesting that this particular assay may be of limited relevance for PM toxicity and effects.

The number and variation of biological endpoints investigated for possible association with acellular OP is strikingly high. Indeed, it may seem unlikely, given the complexity of PM composition, the number of distinct PM sources and the variability in biological effects attributed to PM exposure, that all particle induced effects should be caused by one common triggering mechanism: OP. Indeed, several redox-independent triggering mechanisms for PM toxicity have been described, as discussed later. Nevertheless, virtually all possible effects from particle exposure have to some extent been compared to OP. A general impression is therefore that the search for possible associations between OP and PM toxicity has been performed rather randomly, without clear rational for the choice of biological endpoints included in the respective studies. This possibly reflects a lack of consensus regarding the type of effects that most likely could be caused by direct redox-reactions from particles and particle components, and hence would most likely be associated with acellular OP. The present review may bring some clarification to this issue, as the identified studies appear to show a predominance of positive associations between OP and redox-related outcomes in vitro, and asthma/wheeze in humans. The lack of consistent association between OP and other PM-induced effects could be related to study designs and inherent weaknesses in common techniques for OP assessment, to cell-free assays being unable to recreate in vivo conditions, or simply because many effects of particle exposure are triggered through redox-independent mechanisms. Most likely, the lack of consistency arises from a combination of the aforementioned factors.

### 4.1. Considerations Regarding the Methods Used to Measure OP

A detailed discussion of the strengths and weaknesses of the various acellular assays used for OP measurements was beyond the scope of the present paper, and has been extensively covered elsewhere [26,85,86,87,88,89]. A key problem with most OP assays is that they are not precise enough and do not sufficiently discriminate between different reactive species, and different assays detect different species and with varying efficiency (Table 1). Importantly, there is considerable difference in reactivity, longevity and potential to inflict damage to biomolecules between different types of ROS. Helmut Sies, who pioneered the work on oxidative stress, has emphasized that “Simply to talk of ‘exposing cells or organisms to oxidative stress’ should clearly be discouraged. Instead, the exact molecular condition employed to change the redox balance of a given system is what is important” [90]. Unfortunate, such critical information on “the exact molecular condition employed” when exposing cells, animals or humans to PM cannot be provided by the OP assays currently in use. The different assays also use different units for expressing redox properties, as emphasized by others [87]. Thus, it could be argued that OP assays provide neither quantitative nor qualitative measurements. As consequence, OP measurements of the same particle types obtained by different techniques may report considerably different results. This could likely contribute to the lack of correlation with biological effects and explain some of the discrepancies reported from different studies. In this context introduction of standardized reference particles could be a way forward to increase the comparability of OP measurements reported across different studies.

More specific problems are related to some of the assays. The deoxyribose assay has for instance been highly controversial due to some serious caveats, which may lead to misinterpretation of the findings [86]. Assays such as the DTT test cannot differentiate between redox cycling and “simple” one/two electron oxidations. It has also recently been suggested that quinones and transition metals may catalyze DTT oxidation to an extent not relevant for their effects on biological macromolecule, thus confounding the DTT assay [26].

The only method that provides direct quantification of radical species is ESR (or EPR). ESR can detect and quantify persistent radicals including quinones on combustion particles and surface radicals on quartz, while short-lived radicals like O_2_^●−^ and ^●^OH can be measured more indirectly using a spin trap such as DMPO. However, low sensitivity of ESR may be a potential problem [87]. Moreover, ESR with DMPO is often performed in presence of H_2_O_2_ as substrate for ^●^OH formation, amongst others by Fenton-reactive transition metals. It has been argued that this mimics the conditions during phagocytosis, where engulfed dust particles will be in contact with H_2_O_2_ [29]. Whether addition of H_2_O_2_ is relevant for effects on non-phagocytic cells are less clear, as H_2_O_2_ otherwise would not be present in abundancy under normal physiological condition.

Another central question is there whether acellular OP assays can reflect the complexity of in vivo biological conditions. OP tends to be measured under very artificial conditions, often in simple buffers solutions, that do not reflect the composition of the extracellular fluids in the respiratory tract or elsewhere, or the intracellular milieu. Observing that particles or particle components are able to oxidize a single available substrate in a test tube does not necessarily reflect that critical cellular components would be oxidized under real-life conditions when other competing substrates are present, including (but not restricted to) extracellular and intracellular antioxidant. Moreover, the majority of studies reviewed in this paper appear to have performed OP assays at pH 7.4 (if given at all), which is in the pH range of cell growth media and extracellular compartments. Lung surfactant is more acidic and may be in the range of pH 6.6–7.1 [91], and phagocytosed particles encounter a pH of 4.5–5.0 within the lysosomes [92]. It remains unclear whether increased acidity would alter the relative OP of particles. However, the catalytic redox rates of quinones is affected by pH [93,94]. It has also been reported that the DTT assay may be affected by the pH of the assay buffer, producing not only quantitatively, but also qualitatively different outcomes (different rank-order) when assessing the OP of multiple particles in different simulated lung fluids [95]. A number of assay conditions may potentially differ from real-life scenarios, including relevant competition kinetics and sufficient electron donor concentrations to allow proper redox cycling. Thus, there is a need to link what transpires under acellular artificial conditions with the biological and biochemical processes and pathways involved in PM-exposed cells and tissues.

Many studies identified in this review utilized only one concentration of particles for measurements of OP (Tables S1 and S2, Supplementary Materials). However, the concentration-dependent formation of ROS by particles in cell-free systems is not necessarily linear [33,96]. This could potentially hamper proper comparisons between particles of high and low OP, and might be problematic in cases when the concentrations used for assessment of OP differed from those used to assess biological effects.

Finally, it should also be considered that the biological assays, which OP has been compared with, could also be flawed, and therefore not truly reflect particle toxicity [26,97]. Issues related to interference between particles and common cytotoxicity test are frequently debated, and carbon-based particulates may also bind cytokines and other proteins hence interfering with Enzyme-Linked Immunosorbent Assay (ELISA) [98,99,100]. The oxidant probe, DCF, may leak back out of cells and not reflect intracellular ROS, and LDH used for assessment of cytotoxicity can be inactivated by oxidation, potentially making the assay prone to errors when used in experiments with high levels of oxidative stress [101].

### 4.2. Redox-Independent Mechanisms of PM Toxicity

One of the most obvious explanations for why OP was not associated with PM-induced effects in several studies is the possibility that OP was not the key driver of the effects in questions. Indeed, a number of redox-independent triggering mechanisms for particle toxicity have been reported, including specific receptor mediated effects and interference with membrane lipids, as reviewed elsewhere [9,102]. The toxicity of ambient air PM is particularly complex and is in part related to soluble constituents, which includes a multitude of reactive organic chemicals, metal ions and biological material, of which many interacts directly with cellular receptors and other signaling proteins [9,102,103]. Zinc, which mediates its effects by binding to cellular enzymes, has already been mentioned. Moreover, proinflammatory effects of coarse PM have in many cases (although not always) been attributed to the presence of bacterial endotoxins and activation of toll-like receptors [34,35,68,104,105]. Zinc and endotoxin content would not affect the outcome of acellular measurements of OP. Furthermore, organic compounds do not only mediate their effects through oxidative stress. PAHs and dioxin-like compounds are well known to activate the cytosolic aryl hydrocarbon receptor (AhR), which in its classical mode of action regulates the expression of Phase 1 metabolizing enzymes, such as CYP1A1, -1A2 and -1B1. CYP1-mediated metabolism may produce a number of redox-reactive metabolites from parent compounds that are not directly measured in acellular OP assays. AhR also possesses several other physiological functions. Amongst others, it seems to play a central role in the regulation of inflammation and immune responses [106,107]. There is an extensive crosstalk between AhR and the proinflammatory transcription factor NF-κB [108], and AhR may also be directly involved in the transcriptional regulation of proinflammatory genes [109,110,111,112,113,114]. AhR and PAHs also appear to play a central role in CVD, as reviewed elsewhere [115,116,117], providing a potential link between PM exposure and adverse effects. These are other possible components and effects not picked-up by acellular assays for OP.

While both oxidant and non-oxidant mediated triggering mechanisms for PM toxicity exists [9], these different mechanisms may likely follow different dose-response relationships, and their relative importance for the initiation of different PM-induced effects remains unclear. There is a need to clarify whether the levels of ROS produced under abiotic conditions are sufficient to produce the biological effects in question, and whether such concentrations are realistic and relevant. Indeed, the oxidative stress paradigm has been extensively debated in recent years and it has been argued that the levels of exogenous oxidants required to distorting the physiological oxidant-antioxidant balance and cause pathology is order of magnitudes higher than what could be reached under real-life conditions in either health or disease [118]. A central task should therefore be to clarify which triggering mechanisms (oxidant or non-oxidant) are the most important for effects at low PM concentrations, realistic for real-life PM exposure scenarios.

### 4.3. Correlation is not Causation

Any scientist should be well aware that correlations and associations do not confirm causation. Even in the cases when OP appears to be associated with effects, other factors may be the underlying cause. A central question is therefore whether OP could be an indicator of something else?

Sampling of ambient PM before and during the closure and after the reopening of a steel mill in Utah Valley has allowed for examination of the contribution of metal-rich particulates to the toxicity PM. Some studies on Utah Valley PM have indicated a possible association between OP (deoxy ribose assay) and inflammatory responses [28,61]. Both metal chelator (deferroxamine) and antioxidant (DMTU and DMSO) treatment blocked acellular ROS generation and production of proinflammatory cytokines in BEAS-2B cells pointing to a possible role of redox active transition metals [28]. However, other studies do not support a clear causal relation between the OP and biological effects of Utah Valley PM [27,31]. Of notice, Utah Valley PM contained high levels of zinc (twice the level of copper and six-fold higher than iron), and the zinc-level was also considerably reduced by the metal-chelator treatment [31]. Studies by Samet and colleagues suggest that zinc could be an important contributor to the proinflammatory effects of metal rich PM [119]. In contrast to metals such as iron and copper, zinc is not redox active but may elicit effects by inhibiting protein phosphatases [120,121]. In fact, studies using metal ions suggest that only zinc and not copper or iron ions were capable of inducing IL-6 and CXL8 responses in the BEAS-2B cells [121,122]. Thus, reductions in proinflammatory effects of Utah Valley PM observed after metal-chelation treatment, could at least in part be due to a reduction in zinc and other redox-inactive constituents, rather than the corresponding reduction in redox active metals and OP. The Utah Valley studies underscores that correlation or association between two observations does not necessarily imply causality, and that other covariates may be more important to the biological effects than OP. Indeed, strong associations have been reported between zinc concentrations in PM and OP measured by different assays, despite the fact that zinc is not detected by OP assays [58].

In striking resemblance of the Utah Valley PM studies, a study on metal-rich PM_2.5_ from the German smelter area Hettstedt and control PM_2.5_ from a non-industrialized area found that the metal-rich Hettstedt-PM_2.5_ induced stronger pulmonary inflammation in healthy volunteers [62]. Although only two particle types do not allow for correlation analysis, the higher proinflammatory effect of metal-rich PM_2.5_ was accompanied by a higher acellular OP (ESR with H_2_O_2_ and DMPO). It was therefore suggested that the higher concentration of redox-active metals in Hettstedt-PM_2.5_ could be responsible for its increased inflammogenicity [62]. However, the most abundant metal in Hettstedt-PM_2.5_ was zinc (six-fold higher than copper and seven-fold higher than iron). It seems possible that the inflammatory effect of the metal-rich Hettstedt-PM_2.5_, at least in part, could have been due to the high zinc content and not necessarily the OP of the particles, as in the case of the Utah Valley PM.

As already discussed, PAHs are another group of PM constituents considered to be among the potential key drivers of PM toxicity [9,115], which are not measured by acellular OP assays. As with zinc, the PAH content of PM may also be strongly associated with OP [58]. A likely explanation for this is that many redox-active organic chemicals such as quinones and oxy-PAHs are produced alongside PAHs during combustion processes, or may be derived directly from PAHs, for instance through photooxidation. In this aspect, it is interesting to note that OP measured by the DTT assay may be statistically significantly correlated with CYP1A1 expression [58,60]. AhR, the key regulator of CYP1A1, is also the major cellular sensor of PAHs and other nonpolar compounds, but reacts poorly to the more polar quinones and oxy-PAHs. Thus, OP measured by the DTT assay is likely associated with at least some outcomes indirectly, due to PM-bound PAHs co-varying with quinones, oxy-PAHs or other redox active organics detected by DTT.

Whether reported associations between OP and effects are truly due to the direct redox properties of PM or more indirectly through correlation between OP and other redox-independent PM properties remains to be determined. Nevertheless, several epidemiological studies have reported that effects in humans were stronger associations OP than PM mass. This suggests that particle composition may be important for the effects, as pointed out by Strak and colleagues [83]. A series of studies from Ontario, Canada, show that OP (GSH- but not AA-depletion) modified the impact of respiratory illness, myocardial infarctions and birth outcomes [79,80,82]. Thus, it would be of particular interest to clarify whether OP or PM components correlating with OP, could explain some of the between-city heterogeneity in risk estimates reported for PM exposure [123].

### 4.4. The Relative Toxicity of Particles Varies across Different Endpoints and Target Cells or Tissues

Another important aspect is that the relative toxicity of particles may vary depending on endpoints and cellular targets. The proinflammmatory potential of different particles does not necessarily correlate with their ability to induce other cellular responses including intracellular ROS formation and cytotoxicity. Even for a specific type of effects (i.e., inflammatory responses) the rank order of potency among different particle types may vary between different cell types and between in vitro cell culture tests and in vivo animal studies. This has been reported in a number of studies on ambient air PM as well as mineral particles and nanomaterials [105,124,125,126,127,128,129,130,131,132,133,134]. These observations not only suggest the existence of a variety of triggering mechanisms for particle toxicity and that different particle characteristics may act as critical determinants for different toxicological effects, but also that triggering mechanisms may vary between different cell types and target organs. Based on this, it seems unlikely that a single determinant such as OP could be a useful predictor of all particle-induced outcomes.

In extension of the above, Donaldson et al. [97] raised another important issue. In a review on the limitation of intracellular oxidative stress as a predictor of particle toxicity they pointed out that the three pathogenic particle types PM_10_, asbestos and quartz have all been reported to induce comparable effects on intracellular ROS formation in vitro, but in real-life exposure scenarios they induce diverse pathologies [97]. Similarly, it could be argued that also OP in cell-free/abiotic systems is incapable of discriminating between classes of particles with different pathological effects.

## 5. Conclusions

The collective evidence suggest that there is limited or no clear evidence of association between individual OP assays and inflammation, non-oxidative DNA-damage and cytotoxicity in experimental studies (in vitro), as well as COPD, CVD, mortality and biomarkers of inflammation and oxidative stress in epidemiological and clinical studies in humans. However, some evidence exists for positive reproducible associations between OP assays and redox responses including intracellular ROS, oxidative damage and antioxidant responses in vitro, and between OP measured by the DTT assay and asthma/wheeze in humans. The limited association with several outcomes could in part be due to the low precision and considerable variation in the ability to detect different reactive species among the OP assays applied, but also due to the inability of such acellular assays to simulate what occurs under biological conditions in real-life. However, it most likely also reflects that other redox-independent triggering mechanisms are important for PM-induced effects. Clarifying these issues will be important to advance the field.

Finally, it should be appropriate to repeat that correlation alone is insufficient to provide causality to a hypothesis, and that a lack of correlation is equally insufficient for its falsification. There may be a number of reasons why OP correlates with a biological effect in one study but not in another. Additional evidence must therefore also be considered. However, the lack of a consistent association with a broad range of biological effects in cells, animals and humans suggests that current assays for OP of particles in cell-free/abiotic systems may have limited value in predicting toxicity of PM and combustion particles.

## Figures and Tables

**Table 1:** Assays for detection of the oxidative potential (OP) in cell-free/abiotic systems.

Assay	Species Detected
AA-depletion	Used to measure oxidative potential of transition metals (^●^OH from H_2_O_2_). Interacts with several other reactive species.
GSH-depletion	Most ROS as well as peroxides, alkenals, protein disulfides and sulfenic acids
Congo Red	Hydroxylic, peroxide and hydroperoxide radicals
DCF (DCFH-DA)	^●^NO_2_, ^●^OH, ONOO^−^, peroxyl, aloxyl and carbon-centered radicals, peroxides. Can be used to measure H_2_O_2_ in presence of a peroxidase catalyst (HRP). Prone to photooxidation.
2-deoxyribose	^●^OH and ^●^OH-like species (used as a simple and inexpensive substitute for ESR).
DHE	Can be specific for O_2_^●−^ (require separation of products by HPLC). Interacts with several other reactive species.
DTT	Diverse range of free radicals and reactive species. Reduced by transition metals and quinones, in PM.
ESR (or EPR)	ESR with DMPO as spin-trap measures production of ^●^OH, and is often used in combination with H_2_O_2_. ESR without spin-trapping can be used to measure surface radicals on particles (measures unpaired electrons).
Luminol	HOCl, H_2_O_2_ and ONOO^−^

Abbreviations: AA—ascorbic acid; DCF—dichlorofluorescin; DCFH-DA—dichlorodihydrofluorescein diacetate; DHE—dihydroethidium; DMPO—5,5-dimethyl-pyrroline N-oxide; EPR—electron paramagnetic resonance; ESR—electron spin resonance; DTT—dithiothreitol; GSH—reduced glutathione; H_2_O_2_—hydrogen peroxide; HOCl—hypochlorous acid; HPLC—High-performance liquid chromatography; MDA—malondialdehyde; ^●^NO_2_—nitrogen dioxide; O_2_^●−^—superoxide anion; ^●^OH—hydroxyl radical; ONOO^—^peroxynitrite; PM—particulate matter; ROS—reactive oxygen species.

## Supplementary Materials

Supplementary materials can be found at https://www.mdpi.com/1422-0067/20/19/4772/s1.

## References

[1] Borm P.J. (2002). Particle toxicology: From coal mining to nanotechnology. Inhal. Toxicol..

[2] Grahame T.J., Klemm R., Schlesinger R.B. (2014). Public health and components of particulate matter: The changing assessment of black carbon. J. Air Waste Manag. Assoc..

[3] Kelly F.J., Fussell J.C. (2011). Air pollution and airway disease. Clin. Exp. Allergy.

[4] Kim K.H., Kabir E., Kabir S. (2015). A review on the human health impact of airborne particulate matter. Environ. Int..

[5] Landrigan P.J., Fuller R., Acosta N.J.R., Adeyi O., Arnold R., Basu N.N., Balde A.B., Bertollini R., Bose-O’Reilly S., Boufford J.I. (2018). The Lancet Commission on pollution and health. Lancet.

[6] Landrigan P.J. (2017). Air pollution and health. Lancet. Public Health.

[7] Salvi S., Holgate S.T. (1999). Mechanisms of particulate matter toxicity. Clin. Exp. Allergy.

[8] Donaldson K., Stone V., Seaton A., MacNee W. (2001). Ambient particle inhalation and the cardiovascular system: Potential mechanisms. Environ. Health Perspect..

[9] Øvrevik J., Refsnes M., Lag M., Holme J.A., Schwarze P.E. (2015). Activation of Proinflammatory Responses in Cells of the Airway Mucosa by Particulate Matter: Oxidant- and Non-Oxidant-Mediated Triggering Mechanisms. Biomolecules.

[10] Brits E., Schoeters G., Verschaeve L. (2004). Genotoxicity of PM10 and extracted organics collected in an industrial, urban and rural area in Flanders, Belgium. Environ. Res..

[11] Gualtieri M., Rigamonti L., Galeotti V., Camatini M. (2005). Toxicity of tire debris extracts on human lung cell line A549. Toxicol. In Vitro.

[12] Gualtieri M., Øvrevik J., Mollerup S., Longhin E., Dahlman H.J., Camatini M., Holme J.A. (2011). Airborne urban particles (Milan winter- PM_2.5_) cause mitotic arrest and cell death: Effects on DNA, mitochondria, AhR binding and spindle organization. Mutat. Res..

[13] Longhin E., Holme J.A., Gutzkow K.B., Arlt V.M., Kucab J.E., Camatini M., Gualtieri M. (2013). Cell cycle alterations induced by urban PM_2.5_ in bronchial epithelial cells: Characterization of the process and possible mechanisms involved. Part. Fibre Toxicol..

[14] Donaldson K., Stone V., Borm P.J.A., Jimenez L.A., Gilmour P.S., Schins R.P.F., Knaapen A.M., Rahman I., Faux S.P., Brown D.M. (2003). Oxidative stress and calcium signaling in the adverse effects of environmental particles (PM_10_). Free Radic. Biol. Med..

[15] Li N., Xia T., Nel A.E. (2008). The role of oxidative stress in ambient particulate matter-induced lung diseases and its implications in the toxicity of engineered nanoparticles. Free Radic. Biol. Med..

[16] Moller P., Danielsen P.H., Karottki D.G., Jantzen K., Roursgaard M., Klingberg H., Jensen D.M., Christophersen D.V., Hemmingsen J.G., Cao Y. (2014). Oxidative stress and inflammation generated DNA damage by exposure to air pollution particles. Mutat. Res. Rev. Mutat. Res..

[17] Nel A., Xia T., Madler L., Li N. (2006). Toxic potential of materials at the nanolevel. Science.

[18] Shi X., Castranova V., Halliwell B., Vallyathan V. (1998). Reactive oxygen species and silica-induced carcinogenesis. J. Toxicol. Environ. Health B Crit. Rev..

[19] Shukla A., Gulumian M., Hei T.K., Kamp D., Rahman Q., Mossman B.T. (2003). Multiple roles of oxidants in the pathogenesis of asbestos-induced diseases. Free Radic. Biol. Med..

[20] Ayres J.G., Borm P., Cassee F.R., Castranova V., Donaldson K., Ghio A., Harrison R.M., Hider R., Kelly F., Kooter I.M. (2008). Evaluating the toxicity of airborne particulate matter and nanoparticles by measuring oxidative stress potential--a workshop report and consensus statement. Inhal. Toxicol..

[21] Borm P.J., Kelly F., Kunzli N., Schins R.P., Donaldson K. (2007). Oxidant generation by particulate matter: From biologically effective dose to a promising, novel metric. Occup. Environ. Med..

[22] Kunzli N., Mudway I.S., Gotschi T., Shi T., Kelly F.J., Cook S., Burney P., Forsberg B., Gauderman J.W., Hazenkamp M.E. (2006). Comparison of oxidative properties, light absorbance, total and elemental mass concentration of ambient PM_2.5_ collected at 20 European sites. Environ. Health Perspect..

[23] Sarnat S.E., Chang H.H., Weber R.J. (2016). Ambient PM_2.5_ and Health: Does PM_2.5_ Oxidative Potential Play a Role?. Am. J. Respir. Crit. Care Med..

[24] Bates J.T., Fang T., Verma V., Zeng L., Weber R.J., Tolbert P.E., Abrams J.Y., Sarnat S.E., Klein M., Mulholland J.A. (2019). Review of Acellular Assays of Ambient Particulate Matter Oxidative Potential: Methods and Relationships with Composition, Sources, and Health Effects. Environ. Sci. Technol..

[25] Nathan C., Cunningham-Bussel A. (2013). Beyond oxidative stress: An immunologist’s guide to reactive oxygen species. Nat. Rev. Immunol..

[26] Forman H.J., Finch C.E. (2018). A critical review of assays for hazardous components of air pollution. Free Radic. Biol. Med..

[27] Frampton M.W., Ghio A.J., Samet J.M., Carson J.L., Carter J.D., Devlin R.B. (1999). Effects of aqueous extracts of PM(10) filters from the Utah valley on human airway epithelial cells. Am. J. Physiol..

[28] Ghio A.J., Stonehuerner J., Dailey L.A., Carter J.D. (1999). Metals associated with both the water-soluble and insoluble fractions of an ambient air pollution particle catalyze an oxidative stress. Inhal. Toxicol..

[29] Van Maanen J.M.S., Borm P.J.A., Knaapen A., van Herwijnen M., Schilderman P.A.E.L., Smith K.R., Aust A.E., Tomatis M., Fubini B. (1999). In vitro effects of coal fly ashes: Hydroxyl radical generation, iron release, and DNA damage and toxicity in rat lung epithelial cells. Inhal. Toxicol..

[30] Dellinger B., Pryor W.A., Cueto R., Squadrito G.L., Hegde V., Deutsch W.A. (2001). Role of free radicals in the toxicity of airborne fine particulate matter. Chem. Res. Toxicol..

[31] Molinelli A.R., Madden M.C., McGee J.K., Stonehuerner J.G., Ghio A.J. (2002). Effect of metal removal on the toxicity of airborne particulate matter from the Utah Valley. Inhal. Toxicol..

[32] Li N., Sioutas C., Cho A., Schmitz D., Misra C., Sempf J., Wang M., Oberley T., Froines J., Nel A. (2003). Ultrafine particulate pollutants induce oxidative stress and mitochondrial damage. Environ. Health Perspect..

[33] Shi T., Knaapen A.M., Begerow J., Birmili W., Borm P.J., Schins R.P. (2003). Temporal variation of hydroxyl radical generation and 8-hydroxy-2’-deoxyguanosine formation by coarse and fine particulate matter. Occup. Environ. Med..

[34] Salonen R.O., Halinen A.I., Pennanen A.S., Hirvonen M.R., Sillanpaa M., Hillamo R., Shi T., Borm P., Sandell E., Koskentalo T. (2004). Chemical and in vitro toxicologic characterization of wintertime and springtime urban-air particles with an aerodynamic diameter below 10 microm in Helsinki. Scand. J. Work Environ. Health.

[35] Schins R.P., Lightbody J.H., Borm P.J., Shi T., Donaldson K., Stone V. (2004). Inflammatory effects of coarse and fine particulate matter in relation to chemical and biological constituents. Toxicol. Appl. Pharm..

[36] Beck-Speier I., Dayal N., Karg E., Maier K.L., Schumann G., Schulz H., Semmler M., Takenaka S., Stettmaier K., Bors W. (2005). Oxidative stress and lipid mediators induced in alveolar macrophages by ultrafine particles. Free. Radic. Biol. Med..

[37] De Vizcaya-Ruiz A., Gutierrez-CastiBarbier O., Usachenko J.L., Uribe-Ramirez M., Cebrian M.E., Mugica-Alvarez V., Sepulveda J., Rosas I., Salinas E., Garcia-Cuellar C. (2006). Characterization and in vitro biological effects of concentrated particulate matter from Mexico City. Atmos. Environ..

[38] Shi T., Duffin R., Borm P.J., Li H., Weishaupt C., Schins R.P. (2006). Hydroxyl-radical-dependent DNA damage by ambient particulate matter from contrasting sampling locations. Environ. Res..

[39] Leonard S.S., Castranova V., Chen B.T., Schwegler-Berry D., Hoover M., Piacitelli C., Gaughan D.M. (2007). Particle size-dependent radical generation from wildland fire smoke. Toxicology.

[40] Repine J.E., Reiss O.K., Elkins N., Chughtai A.R., Smith D.M. (2008). Effects of fine carbonaceous particles containing high and low unpaired electron spin densities on lungs of female mice. Transl. Res..

[41] Verma V., Polidori A., Schauer J.J., Shafer M.M., Cassee F.R., Sioutas C. (2009). Physicochemical and toxicological profiles of particulate matter in Los Angeles during the October 2007 southern California wildfires. Environ. Sci. Technol..

[42] Akhtar U.S., McWhinney R.D., Rastogi N., Abbatt J.P., Evans G.J., Scott J.A. (2010). Cytotoxic and proinflammatory effects of ambient and source-related particulate matter (PM) in relation to the production of reactive oxygen species (ROS) and cytokine adsorption by particles. Inhal. Toxicol..

[43] Wessels A., Birmili W., Albrecht C., Hellack B., Jermann E., Wick G., Harrison R.M., Schins R.P. (2010). Oxidant generation and toxicity of size-fractionated ambient particles in human lung epithelial cells. Environ. Sci. Technol..

[44] Steenhof M., Gosens I., Strak M., Godri K.J., Hoek G., Cassee F.R., Mudway I.S., Kelly F.J., Harrison R.M., Lebret E. (2011). In vitro toxicity of particulate matter (PM) collected at different sites in the Netherlands is associated with PM composition, size fraction and oxidative potential--the RAPTES project. Part. Fibre Toxicol..

[45] Danielsen P.H., Moller P., Jensen K.A., Sharma A.K., Wallin H., Bossi R., Autrup H., Molhave L., Ravanat J.L., Briede J.J. (2011). Oxidative stress, DNA damage, and inflammation induced by ambient air and wood smoke particulate matter in human A549 and THP-1 cell lines. Chem. Res. Toxicol..

[46] Danielsen P.H., Loft S., Jacobsen N.R., Jensen K.A., Autrup H., Ravanat J.L., Wallin H., Moller P. (2010). Oxidative stress, inflammation, and DNA damage in rats after intratracheal instillation or oral exposure to ambient air and wood smoke particulate matter. Toxicol. Sci..

[47] Quintana R., Serrano J., Gomez V., de Foy B., Miranda J., Garcia-Cuellar C., Vega E., Vazquez-Lopez I., Molina L.T., Manzano-Leon N. (2011). The oxidative potential and biological effects induced by PM10 obtained in Mexico City and at a receptor site during the MILAGRO Campaign. Environ. Pollut..

[48] Gerlofs-Nijland M.E., Totlandsdal A.I., Tzamkiozis T., Leseman D.L., Samaras Z., Lag M., Schwarze P., Ntziachristos L., Cassee F.R. (2013). Cell toxicity and oxidative potential of engine exhaust particles: Impact of using particulate filter or biodiesel fuel blend. Environ. Sci. Technol..

[49] Delfino R.J., Staimer N., Tjoa T., Gillen D.L., Schauer J.J., Shafer M.M. (2013). Airway inflammation and oxidative potential of air pollutant particles in a pediatric asthma panel. J. Expos. Sci. Environ. Epidemiol..

[50] Lu Y., Su S., Jin W., Wang B., Li N., Shen H., Li W., Huang Y., Chen H., Zhang Y. (2014). Characteristics and cellular effects of ambient particulate matter from Beijing. Environ. Pollut..

[51] Badding M.A., Fix N.R., Antonini J.M., Leonard S.S. (2014). A comparison of cytotoxicity and oxidative stress from welding fumes generated with a new nickel-, copper-based consumable versus mild and stainless steel-based welding in RAW 264.7 mouse macrophages. PLoS ONE.

[52] Liu Q., Baumgartner J., Zhang Y., Liu Y., Sun Y., Zhang M. (2014). Oxidative potential and inflammatory impacts of source apportioned ambient air pollution in Beijing. Environ. Sci. Technol..

[53] Velali E., Papachristou E., Pantazaki A., Choli-Papadopoulou T., Planou S., Kouras A., Manoli E., Besis A., Voutsa D., Samara C. (2015). Redox activity and in vitro bioactivity of the water-soluble fraction of urban particulate matter in relation to particle size and chemical composition. Environ. Pollut..

[54] Fox J.R., Cox D.P., Drury B.E., Gould T.R., Kavanagh T.J., Paulsen M.H., Sheppard L., Simpson C.D., Stewart J.A., Larson T.V. (2015). Chemical characterization and in vitro toxicity of diesel exhaust particulate matter generated under varying conditions. Air Qual. Atmos. Health.

[55] Van Den Heuvel R., Staelens J., Koppen G., Schoeters G. (2018). Toxicity of Urban PM10 and Relation with Tracers of Biomass Burning. Int. J. Environ. Res. Public Health.

[56] Van Den Heuvel R., Den Hond E., Govarts E., Colles A., Koppen G., Staelens J., Mampaey M., Janssen N., Schoeters G. (2016). Identification of PM10 characteristics involved in cellular responses in human bronchial epithelial cells (Beas-2B). Environ. Res..

[57] Karavalakis G., Gysel N., Schmitz D.A., Cho A.K., Sioutas C., Schauer J.J., Cocker D.R., Durbin T.D. (2017). Impact of biodiesel on regulated and unregulated emissions, and redox and proinflammatory properties of PM emitted from heavy-duty vehicles. Sci. Total. Environ..

[58] Crobeddu B., Aragao-Santiago L., Bui L.C., Boland S., Baeza Squiban A. (2017). Oxidative potential of particulate matter 2.5 as predictive indicator of cellular stress. Environ. Pollut..

[59] He R.W., Shirmohammadi F., Gerlofs-Nijland M.E., Sioutas C., Cassee F.R. (2018). Pro-inflammatory responses to PM_0.25_ from airport and urban traffic emissions. Sci. Total Environ..

[60] Jaramillo I.C., Sturrock A., Ghiassi H., Woller D.J., Deering-Rice C.E., Lighty J.S., Paine R., Reilly C., Kelly K.E. (2018). Effects of fuel components and combustion particle physicochemical properties on toxicological responses of lung cells. J. Environ. Sci. Health. Part A Toxic/Hazard. Subst. Environ. Eng..

[61] Ghio A.J., Devlin R.B. (2001). Inflammatory lung injury after bronchial instillation of air pollution particles. Am. J. Respir. Crit. Care Med..

[62] Schaumann F., Borm P.J., Herbrich A., Knoch J., Pitz M., Schins R.P., Luettig B., Hohlfeld J.M., Heinrich J., Krug N. (2004). Metal-rich ambient particles (particulate matter 2.5) cause airway inflammation in healthy subjects. Am. J. Respir. Crit. Care Med..

[63] Hogervorst J.G., de Kok T.M., Briede J.J., Wesseling G., Kleinjans J.C., van Schayck C.P. (2006). Relationship between radical generation by urban ambient particulate matter and pulmonary function of school children. J. Toxicol. Environ. Health A.

[64] Tonne C., Yanosky J.D., Beevers S., Wilkinson P., Kelly F.J. (2012). PM mass concentration and PM oxidative potential in relation to carotid intima-media thickness. Epidemiology.

[65] Canova C., Minelli C., Dunster C., Kelly F., Shah P.L., Caneja C., Tumilty M.K., Burney P. (2014). PM10 oxidative properties and asthma and COPD. Epidemiology.

[66] Bates J.T., Weber R.J., Abrams J., Verma V., Fang T., Klein M., Strickland M.J., Sarnat S.E., Chang H.H., Mulholland J.A. (2015). Reactive Oxygen Species Generation Linked to Sources of Atmospheric Particulate Matter and Cardiorespiratory Effects. Environ. Sci. Technol..

[67] Janssen N.A., Strak M., Yang A., Hellack B., Kelly F.J., Kuhlbusch T.A., Harrison R.M., Brunekreef B., Cassee F.R., Steenhof M. (2015). Associations between three specific a-cellular measures of the oxidative potential of particulate matter and markers of acute airway and nasal inflammation in healthy volunteers. Occup. Environ. Med..

[68] Steenhof M., Mudway I.S., Gosens I., Hoek G., Godri K.J., Kelly F.J., Harrison R.M., Pieters R.H., Cassee F.R., Lebret E. (2013). Acute nasal pro-inflammatory response to air pollution depends on characteristics other than particle mass concentration or oxidative potential: The RAPTES project. Occup. Environ. Med..

[69] Steenhof M., Janssen N.A., Strak M., Hoek G., Gosens I., Mudway I.S., Kelly F.J., Harrison R.M., Pieters R.H., Cassee F.R. (2014). Air pollution exposure affects circulating white blood cell counts in healthy subjects: The role of particle composition, oxidative potential and gaseous pollutants—the RAPTES project. Inhal. Toxicol..

[70] Strak M., Janssen N.A., Godri K.J., Gosens I., Mudway I.S., Cassee F.R., Lebret E., Kelly F.J., Harrison R.M., Brunekreef B. (2012). Respiratory health effects of airborne particulate matter: The role of particle size, composition, and oxidative potential-the RAPTES project. Environ. Health Perspect..

[71] Strak M., Hoek G., Steenhof M., Kilinc E., Godri K.J., Gosens I., Mudway I.S., van Oerle R., Spronk H.M., Cassee F.R. (2013). Components of ambient air pollution affect thrombin generation in healthy humans: The RAPTES project. Occup. Environ. Med..

[72] Strak M., Hoek G., Godri K.J., Gosens I., Mudway I.S., van Oerle R., Spronk H.M., Cassee F.R., Lebret E., Kelly F.J. (2013). Composition of PM affects acute vascular inflammatory and coagulative markers—The RAPTES project. PLoS ONE.

[73] Atkinson R.W., Samoli E., Analitis A., Fuller G.W., Green D.C., Anderson H.R., Purdie E., Dunster C., Aitlhadj L., Kelly F.J. (2016). Short-term associations between particle oxidative potential and daily mortality and hospital admissions in London. Int. J. Hyg. Environ. Health.

[74] Yang A., Janssen N.A., Brunekreef B., Cassee F.R., Hoek G., Gehring U. (2016). Children’s respiratory health and oxidative potential of PM_2.5_: The PIAMA birth cohort study. Occup. Environ. Med..

[75] Abrams J.Y., Weber R.J., Klein M., Sarnat S.E., Chang H.H., Strickland M.J., Verma V., Fang T., Bates J.T., Mulholland J.A. (2017). Associations between Ambient Fine Particulate Oxidative Potential and Cardiorespiratory Emergency Department Visits. Environ. Health Perspect..

[76] Fang T., Verma V., Bates J.T., Abrams J., Klein M., Strickland M.J., Sarnat S.E., Chang H.H., Mulholland J.A., Tolbert P.E. (2016). Oxidative potential of ambient water-soluble PM_2.5_ in the southeastern United States: Contrasts in sources and health associations between ascorbic acid (AA) and dithiothreitol (DTT) assays. Atmos. Chem. Phys..

[77] Zhang X., Staimer N., Tjoa T., Gillen D.L., Schauer J.J., Shafer M.M., Hasheminassab S., Pakbin P., Longhurst J., Sioutas C. (2016). Associations between microvascular function and short-term exposure to traffic-related air pollution and particulate matter oxidative potential. Environ. Health.

[78] Maikawa C.L., Weichenthal S., Wheeler A.J., Dobbin N.A., Smargiassi A., Evans G., Liu L., Goldberg M.S., Pollitt K.J. (2016). Particulate Oxidative Burden as a Predictor of Exhaled Nitric Oxide in Children with Asthma. Environ. Health Perspect..

[79] Weichenthal S.A., Lavigne E., Evans G.J., Godri Pollitt K.J., Burnett R.T. (2016). Fine Particulate Matter and Emergency Room Visits for Respiratory Illness. Effect Modification by Oxidative Potential. Am. J. Respir. Crit. Care Med..

[80] Weichenthal S., Lavigne E., Evans G., Pollitt K., Burnett R.T. (2016). Ambient PM_2.5_ and risk of emergency room visits for myocardial infarction: Impact of regional PM_2.5_ oxidative potential: A case-crossover study. Environ. Health.

[81] Weichenthal S., Crouse D.L., Pinault L., Godri-Pollitt K., Lavigne E., Evans G., van Donkelaar A., Martin R.V., Burnett R.T. (2016). Oxidative burden of fine particulate air pollution and risk of cause-specific mortality in the Canadian Census Health and Environment Cohort (CanCHEC). Environ. Res..

[82] Lavigne E., Burnett R.T., Stieb D.M., Evans G.J., Godri Pollitt K.J., Chen H., van Rijswijk D., Weichenthal S. (2018). Fine Particulate Air Pollution and Adverse Birth Outcomes: Effect Modification by Regional Nonvolatile Oxidative Potential. Environ. Health Perspect..

[83] Strak M., Janssen N., Beelen R., Schmitz O., Vaartjes I., Karssenberg D., van den Brink C., Bots M.L., Dijst M., Brunekreef B. (2017). Long-term exposure to particulate matter, NO2 and the oxidative potential of particulates and diabetes prevalence in a large national health survey. Environ. Int..

[84] Liu L., Urch B., Szyszkowicz M., Evans G., Speck M., Van Huang A., Leingartner K., Shutt R.H., Pelletier G., Gold D.R. (2018). Metals and oxidative potential in urban particulate matter influence systemic inflammatory and neural biomarkers: A controlled exposure study. Environ. Int..

[85] Charrier J.G., Anastasio C. (2012). On dithiothreitol (DTT) as a measure of oxidative potential for ambient particles: Evidence for the importance of soluble transition metals. Atmos. Chem. Phys..

[86] Genaro-Mattos T.C., Dalvi L.T., Oliveira R.G., Ginani J.S., Hermes-Lima M. (2009). Reevaluation of the 2-deoxyribose assay for determination of free radical formation. Biochim. Biophys. Acta.

[87] Hedayat F., Stevanovic S., Miljevic B., Bottle S., Ristovski Z.D. (2014). Review-evaluating the molecular assays for measuring the oxidative potential of particulate matter. Chem. Ind. Chem. Engin. Quart..

[88] Jones D.P. (2002). Redox potential of GSH/GSSG couple: Assay and biological significance. Methods Enzym..

[89] Myhre O., Andersen J.M., Aarnes H., Fonnum F. (2003). Evaluation of the probes 2’,7’-dichlorofluorescin diacetate, luminol, and lucigenin as indicators of reactive species formation. Biochem. Pharm..

[90] Sies H. (2015). Oxidative stress: A concept in redox biology and medicine. Redox Biol..

[91] Ng A.W., Bidani A., Heming T.A. (2004). Innate host defense of the lung: Effects of lung-lining fluid pH. Lung.

[92] Mindell J.A. (2012). Lysosomal acidification mechanisms. Annu. Rev. Physiol..

[93] Isaacs N., van Eldik R. (1997). A mechanistic study of the reduction of quinones by ascorbic acid. J. Chem. Soc. Perkin Trans. 2.

[94] Guin P.S., Das S., Mandal P.C. (2011). Electrochemical Reduction of Quinones in Different Media: A Review. Int. J. Electrochem..

[95] Calas A., Uzu G., Martins J.M.F., Voisin D., Spadini L., Lacroix T., Jaffrezo J.L. (2017). The importance of simulated lung fluid (SLF) extractions for a more relevant evaluation of the oxidative potential of particulate matter. Sci. Rep..

[96] Hussain S., Boland S., Baeza-Squiban A., Hamel R., Thomassen L.C., Martens J.A., Billon-Galland M.A., Fleury-Feith J., Moisan F., Pairon J.C. (2009). Oxidative stress and proinflammatory effects of carbon black and titanium dioxide nanoparticles: Role of particle surface area and internalized amount. Toxicology.

[97] Donaldson K., Borm P.J., Castranova V., Gulumian M. (2009). The limits of testing particle-mediated oxidative stress in vitro in predicting diverse pathologies; relevance for testing of nanoparticles. Part. Fibre Toxicol..

[98] Kocbach A., Totlandsdal A.I., Lag M., Refsnes M., Schwarze P.E. (2008). Differential binding of cytokines to environmentally relevant particles: A possible source for misinterpretation of in vitro results?. Toxicol. Lett..

[99] Kroll A., Pillukat M.H., Hahn D., Schnekenburger J. (2012). Interference of engineered nanoparticles with in vitro toxicity assays. Arch. Toxicol..

[100] Kong B., Seog J.H., Graham L.M., Lee S.B. (2011). Experimental considerations on the cytotoxicity of nanoparticles. Nanomed. (Lond. Engl.).

[101] Buchanan J.D., Armstrong D.A. (1976). Free radical inactivation of lactate dehydrogenase. Int. J. Radiat. Biol. Relat. Stud. Phys. Chem. Med..

[102] Fariss M.W., Gilmour M.I., Reilly C.A., Liedtke W., Ghio A.J. (2013). Emerging mechanistic targets in lung injury induced by combustion-generated particles. Toxicol. Sci..

[103] Akopian A.N., Fanick E.R., Brooks E.G. (2016). TRP channels and traffic-related environmental pollution-induced pulmonary disease. Semin. Immunopathol..

[104] Becker S., Fenton M.J., Soukup J.M. (2002). Involvement of microbial components and Toll-like receptors 2 and 4 in cytokine responses to air pollution particles. Am. J. Respir. Cell Mol. Biol..

[105] Gualtieri M., Øvrevik J., Holme J.A., Perrone M.G., Bolzacchini E., Schwarze P.E., Camatini M. (2010). Differences in cytotoxicity versus pro-inflammatory potency of different PM fractions in human epithelial lung cells. Toxicol. In Vitro.

[106] Fardel O. (2013). Cytokines as molecular targets for aryl hydrocarbon receptor ligands: Implications for toxicity and xenobiotic detoxification. Expert. Opin. Drug Metab. Toxicol..

[107] Stockinger B., Di M.P., Gialitakis M., Duarte J.H. (2014). The aryl hydrocarbon receptor: Multitasking in the immune system. Annu. Rev. Immunol..

[108] Tian Y., Rabson A.B., Gallo M.A. (2002). Ah receptor and NF-kappaB interactions: Mechanisms and physiological implications. Chem. Biol. Interact..

[109] Chen P.H., Chang H., Chang J.T., Lin P. (2012). Aryl hydrocarbon receptor in association with RelA modulates IL-6 expression in non-smoking lung cancer. Oncogene.

[110] N’Diaye M., Le F.E., Lagadic-Gossmann D., Corre S., Gilot D., Lecureur V., Monteiro P., Rauch C., Galibert M.D., Fardel O. (2006). Aryl hydrocarbon receptor- and calcium-dependent induction of the chemokine CCL1 by the environmental contaminant benzo[a]pyrene. J. Biol. Chem..

[111] Podechard N., Lecureur V., Le Ferrec E., Guenon I., Sparfel L., Gilot D., Gordon J.R., Lagente V., Fardel O. (2008). Interleukin-8 induction by the environmental contaminant benzo(a)pyrene is aryl hydrocarbon receptor-dependent and leads to lung inflammation. Toxicol. Lett..

[112] Vogel C.F., Sciullo E., Matsumura F. (2007). Involvement of RelB in aryl hydrocarbon receptor-mediated induction of chemokines. Biochem. Biophys. Res. Commun..

[113] Vogel C.F., Matsumura F. (2009). A new cross-talk between the aryl hydrocarbon receptor and RelB, a member of the NF-kappaB family. Biochem. Pharm..

[114] Wright C.W., Duckett C.S. (2009). The aryl hydrocarbon nuclear translocator alters CD30-mediated NF-kappaB-dependent transcription. Science.

[115] Holme J.A., Brinchmann B.C., Refsnes M., Lag M., Ovrevik J. (2019). Potential role of polycyclic aromatic hydrocarbons as mediators of cardiovascular effects from combustion particles. Environ. Health.

[116] Holme J.A., Brinchmann B.C., Le Ferrec E., Lagadic-Gossmann D., Ovrevik J. (2019). Combustion Particle-Induced Changes in Calcium Homeostasis: A Contributing Factor to Vascular Disease?. Cardiovasc. Toxicol..

[117] Yi T., Wang J., Zhu K., Tang Y., Huang S., Shui X., Ding Y., Chen C., Lei W. (2018). Aryl Hydrocarbon Receptor: A New Player of Pathogenesis and Therapy in Cardiovascular Diseases. Biomed. Res. Int..

[118] Levonen A.L., Hill B.G., Kansanen E., Zhang J., Darley-Usmar V.M. (2014). Redox regulation of antioxidants, autophagy, and the response to stress: Implications for electrophile therapeutics. Free Radic. Biol. Med..

[119] Kodavanti U.P., Schladweiler M.C.J., Ledbetter A.D., Hauser R., Christiani D.C., Samet J.M., McGee J., Richards J.H., Costa D.L. (2002). Pulmonary and systemic effects of zinc-containing emission particles in three rat strains: Multiple exposure scenarios. Toxicol. Sci..

[120] Tal T.L., Graves L.M., Silbajoris R., Bromberg P.A., Wu W., Samet J.M. (2006). Inhibition of protein tyrosine phosphatase activity mediates epidermal growth factor receptor signaling in human airway epithelial cells exposed to Zn(2+). Toxicol. Appl. Pharm..

[121] Samet J.M., Graves L.M., Quay J., Dailey L.A., Devlin R.B., Ghio A.J., Wu W., Bromberg P.A., Reed W. (1998). Activation of MAPKs in human bronchial epithelial cells exposed to metals. Ajp Lung Cell. Mol. Physiol..

[122] Øvrevik J., Lag M., Holme J.A., Schwarze P.E., Refsnes M. (2009). Cytokine and chemokine expression patterns in lung epithelial cells exposed to components characteristic of particulate air pollution. Toxicology.

[123] Baxter L.K., Crooks J.L., Sacks J.D. (2017). Influence of exposure differences on city-to-city heterogeneity in PM_2.5_-mortality associations in US cities. Environ. Health.

[124] Amatullah H., North M.L., Akhtar U.S., Rastogi N., Urch B., Silverman F.S., Chow C.W., Evans G.J., Scott J.A. (2012). Comparative cardiopulmonary effects of size-fractionated airborne particulate matter. Inhal. Toxicol..

[125] Becker S., Dailey L.A., Soukup J.M., Grambow S.C., Devlin R.B., Huang Y.C. (2005). Seasonal variations in air pollution particle-induced inflammatory mediator release and oxidative stress. Environ. Health Perspect..

[126] Happo M.S., Salonen R.O., Halinen A.I., Jalava P.I., Pennanen A.S., Kosma V.M., Sillanpaa M., Hillamo R., Brunekreef B., Katsouyanni K. (2007). Dose and time dependency of inflammatory responses in the mouse lung to urban air coarse, fine, and ultrafine particles from six European cities. Inhal. Toxicol..

[127] Cho W.S., Duffin R., Bradley M., Megson I.L., MacNee W., Lee J.K., Jeong J., Donaldson K. (2013). Predictive value of in vitro assays depends on the mechanism of toxicity of metal oxide nanoparticles. Part. Fibre Toxicol..

[128] Cho S.H., Tong H., McGee J.K., Baldauf R.W., Krantz Q.T., Gilmour M.I. (2009). Comparative toxicity of size-fractionated airborne particulate matter collected at different distances from an urban highway. Environ. Health Perspect..

[129] Gilmour M.I., McGee J., Duvall R.M., Dailey L., Daniels M., Boykin E., Cho S.H., Doerfler D., Gordon T., Devlin R.B. (2007). Comparative toxicity of size-fractionated airborne particulate matter obtained from different cities in the United States. Inhal. Toxicol..

[130] Refsnes M., Hetland R.B., Øvrevik J., Sundfor I., Schwarze P.E., Låg M. (2006). Different particle determinants induce apoptosis and cytokine release in primary alveolar macrophage cultures. Part. Fibre Toxicol..

[131] Iskandar A., Andersen Z.J., Bonnelykke K., Ellermann T., Andersen K.K., Bisgaard H. (2012). Coarse and fine particles but not ultrafine particles in urban air trigger hospital admission for asthma in children. Thorax.

[132] Øvrevik J., Myran T., Refsnes M., Lag M., Becher R., Hetland R.B., Schwarze P.E. (2005). Mineral particles of varying composition induce differential chemokine release from epithelial lung cells: Importance of physico-chemical Characteristics. Ann. Occup. Hyg..

[133] Samet J.M., Graff D., Berntsen J., Ghio A.J., Huang Y.C., Devlin R.B. (2007). A comparison of studies on the effects of controlled exposure to fine, coarse and ultrafine ambient particulate matter from a single location. Inhal. Toxicol..

[134] Lu S., Duffin R., Poland C., Daly P., Murphy F., Drost E., MacNee W., Stone V., Donaldson K. (2009). Efficacy of simple short-term in vitro assays for predicting the potential of metal oxide nanoparticles to cause pulmonary inflammation. Environ. Health Perspect..

