# Supplementary material for: Oxidative Potential Versus Biological Effects: A Review on the Relevance of Cell-Free/Abiotic Assays as Predictors of Toxicity from Airborne Particulate Matter
Source: Int J Mol Sci. 2019 Sep 26;20(19):4772. doi: 10.3390/ijms20194772 (PMC6801578; doi:10.3390/ijms20194772)
Supplement: Supplementary file 1 [file ijms-20-04772-s001.pdf]

OXIDATIVE POTENTIAL VERSUS BIOLOGICAL EFFECTS:

A review on the relevance of cell-free/abiotic assays as predictors of toxicity from airborne particulate matter.

SUPPLEMENTARY MATERIALS

Johan Øvrevik

Department of Air Pollution and Noise,
Division of Environmental Medicine,
Norwegian Institute of Public Health,
Oslo, Norway

Department of Biosciences,
Faculty of Mathematics and
Natural Sciences,
University of Oslo, Norway

Correspondence:

Phone: +47 21 07 64 08/E-mail: johan.ovrevik@fhi.no

References to publications cited in the tables are given at the end of the document.

Table S1. Overview of cell culture and animal studies where biological effects of PM and combustion particles have been examined and compared with acellular oxidative capacity

Particles	Ox-capacity (Cell-free)	Conc. (OP)	Biological endpoints	Conc. (Bio-endpoints)	Association (Ox-capacity vs Bio-endpoints)	Reference
Utah Valley PM_{10} (3 samples)	Deoxyribose assay	500 µg/mL (?) (not clearly specified)	*In vitro* cytotoxicity and induction of IL-6 and CXCL8 in BEAS-2B cells	125-500 µg/mL	No apparent association	Frampton et al. 1999
Water soluble and insoluble fractions of Utah Valley PM_{10} (2 samples)	Deoxyribose assay	500 µg/mL (?) (not clearly specified)	*In vitro* CXCL8 release in BEAS-2B cells, *in vivo* inflammation in rat lungs (PMN and total protein in BALF)	*In vitro*: 500 µg/well in 12 well plate<br><br>*In vivo*: 100-1000 µg/rat	Possible association with both endpoints	Ghio et al. 1999
8 coal fly ashes, crystalline silica, TiO2 and coal dust (11 samples)	ESR with and without H_2O_2 and DMPO	30 mg/mL	*In vitro* cytotoxicity (MTT) and 8-oxo-dG in RLE cells.	Cytotox: not specified LC50 = 0.5-5.1 mg/mL<br><br>8-oxo-dG: LC50 conc. 40 cm^2/mL and $5x10^7$ particles	Statistical significant correlation with 8-oxo-dG, but not with cytotoxicity<br><br>*Note: Correlation apparently obtained between OH•-formation at equal mass and 8-oxo-dG at equal surface area. No correlation with 8-oxo-dG at equal particle number or LC50 conc. TiO_2 (no OH• but 2nd most potent inducer of 8-oxo-dG) was excluded from the correlation analysis.*	van Maanen et al. 1999
$PM_{2.5}$ from different locations (5 samples)	ESR	-	*In vitro* DNA single strand breaks (comet assay) in IB3-1 and K652 cells	33 µg/mL	No apparent association<br><br>*Note: Antioxidant treatment abolished the DNA damage, hence the authors suggested that PM-radicals could be responsible for the effects*	Dellinger et al. 2001
Utah Valley PM (TSP) treated with metal chelator ± replacement of metals (3 samples)	Deoxyribose assay	1 mg/mL	*In vitro* CXCL8 release in BEAS-2B cells, *in vivo* cytotoxicity and inflammation in rat lungs (LDH and total protein in BALF, neutrophilia, fluid infiltrates, and epithelial thickening in lung sections)	*In vitro*: 62.5-1000 µg/mL<br><br>*In vivo*: 1000 µg/rat	Possible association with *in vitro* and *in vivo* cytotoxicity.<br><br>No apparent association with inflammatory reactions *in vitro* and *in vivo*	Molinelli et al. 2002
PM_{10}, $PM_{2.5}$ and UFP, from different sites and seasons (15 samples)	DTT	5-50 µg/mL	*In vitro* induction of HO-1 expression, glutathione depletion and mitochondrial damage in RAW264.7 cells, and induction of HO-1 in BEAS-2B cells	RAW264.7: 12-100 µg/mL<br><br>BEAS-2B: 50 µg/mL	Strong correlation with HO-1 expression in RAW264.7 cells shown by linear regression (r^2 = 0.97). Apparent association with other endpoints.<br><br>*Note: OP^{DTT} highly correlated with PAH content (r^2 = 0.98)*	Li et al. 2003
PM_{10} and $PM_{2.5}$	ESR with H_2O_2 and DMPO	~0.1-2.5 mg/mL (not clearly specified)	*In vitro* induction of 8-oxo-dG in A549 cells	50 µg/mL	No difference in ability to induce 8-oxo-dG despite considerable variation in acellular oxidative capacity	Shi et al. 2003
PM_{10} (3 samples)	ESR with H_2O_2 and DMPO	200 µg/mL	*In vitro* cytotoxicity and induction of TNF-α, IL-6 and NO in RAW264.7 cells	15-1000 µg/mL	No apparent association	Salonen et al. 2004
Coarse and fine PM from 2 locations (4 samples)	ESR with H_2O_2 and DMPO	~0.18 mg/mL	*In vitro* CXCL8 and TNF-α in human whole blood, *in vivo* inflammation (PMN and TNF-α, MIP-2 and LDH) and glutathione depletion in rat lungs (BALF)	*In vitro*:~11-355 µg/mL (?) (not clearly specified)<br><br>*In vivo*:~32 mg/rat	No apparent association	Schins et al. 2004

Studies marked in green show significant correlation/association between at least one OP-measure and all biological effects assessed, confirmed by statistical analysis. Studies marked in blue show significant correlation/association between at least one OP-measure and one biological effect confirmed by statistical analysis. Studies marked in grey show possible correlations/associations, but lacks confirmation by statistical analysis. Studies marked in red show no significant correlation/association between any OP-measures and any biological effects, confirmed by statistical analysis. Studies marked in white show no apparent correlations/associations between any OP-measures and any biological effects, but lacks confirmation by statistical analysis.

Table S1 (continued). Overview of cell culture and animal studies where biological effects of PM and combustion particles have been examined and compared with acellular oxidative capacity

Particles	Ox-capacity (Cell-free)	Conc. (OP)	Biological endpoints	Conc. (Bio-endpoints)	Association (Ox-capacity vs Bio-endpoints)	Reference
EC, DEP and CB of different sizes (4 samples)	ESR<br><br>Oxidation of methionine	100 µg/mL	*In vitro* oxidative stress (8-isoprostane), intracellular ROS, induction of LTB_4, PGE_2, and AA, and activation of $cPLA_2$ in canine AM	1-32 µg/mL (3-240 cm^2/mL depending on particle type)	Possible association with LTB_4 and 8-isoprostane<br><br>Note: No apparent association with AA, PGE_2, $cPLA_2$, or ROS	Beck-Speier et al. 2005
PM_{10} and $PM_{2.5}$ sampled at different locations and seasons (10 samples)	DTT	Coarse: 40 µg/mL<br><br>Fine: 10 µg/mL	*In vitro* cell death (apoptosis and necrosis) in A549 cells and DNA single strand breaks (comet assay) in THP-1 cells	A549: 80 µg/cm^2<br><br>THP-1: 10 µg/mL	No apparent association	De Vizcaya-Ruiz et al. 2006
PM_{10} and $PM_{2.5}$ sampled at different locations and time points (15 samples)	ESR with H_2O_2 and DMPO	Fine: 95 µg/mL<br><br>Coarse: 80 µg/mL	*In vitro* DNA single strand breaks (comet assay) in A549 cells	20 µg/cm^2	No overall/general association when considering all PM sampled together<br><br>*Note: Correlation obtained when urban and rural PM were analyzed separately implying involvement of other factors*	Shi et al. 2006
PM_{10}, $PM_{2.5}$ and UFP from wildfire smoke	ESR with and without H_2O_2 and DMPO	-<br><br>-	*In vitro* intracellular H_2O_2 production, lipid peroxidation and DNA strand breaks in RAW264.7 cells	100 µg/mL	Claimed association with intracellular H_2O_2 and lipid peroxidation, but no statistical analysis presented	Leonard et al. 2007
Combustion particles (2) with high and low spin densities	ESR	-	*In vitro* particle uptake, LTB4 release and mitochondrial damage in NR8383 cells, *in vivo* mitochondrial damage in AM, nitrotyrosine staining and NOx in lungs of mice	*In vitro*: 10 µg/mL<br><br>*In vivo*: 10 µg/m^3 (6h/day, 4 days)	The high-free radical particle induced more effects *in vitro* and *in vivo* than the low-free radical particle<br><br>*Note: Effects on LTB4 directly proportional with increased uptake of high-free radical particles.*	Repine et al. 2008
$PM_{2.5}$ sampled during/after a wildfire (5 samples)	DTT	-	*In vitro* intracellular ROS in primary rat AM	-	No apparent association	Verma et al. 2009
NIST SRM 1648 and SRM 2975, and Toronto $PM_{2.5}$	DTT	100 or 60 µg/mL	*In vitro* cytotoxicity and CXCL8 release in A549 cells	50-1000 µg/mL	No apparent association	Akhtar et al. 2010
Size-fractionated PM from 4 locations (20 samples)	ESR with H_2O_2 and DMPO	-	*In vitro* cytotoxicity, CXCL8 release and oxidative DNA damage (comet assay with FPG) in A549 cells	100 µg/mL	Weak, but statistically significant association with cytotoxicity (r = 0.366, P < 0.001), CXCL8 (r = 0.336, P < 0.01) and oxidative DNA damage (r = 0.559, P < 0.001)<br><br>*Note: OP of smallest size-fraction not associated with biological effects*	Wessels et al. 2010

Studies marked in green show significant correlation/association between at least one OP-measure and all biological effects assessed, confirmed by statistical analysis. Studies marked in blue show significant correlation/association between at least one OP-measure and one biological effect confirmed by statistical analysis. Studies marked in grey show possible correlations/associations, but lacks confirmation by statistical analysis. Studies marked in red show no significant correlation/association between any OP-measures and any biological effects, confirmed by statistical analysis. Studies marked in white show no apparent correlations/associations between any OP-measures and any biological effects, but lacks confirmation by statistical analysis.

Table S1 (continued). Overview of cell culture and animal studies where biological effects of PM and combustion particles have been examined and compared with acellular oxidative capacity

Particles	Ox-capacity (Cell-free)	Conc. (OP)	Biological endpoints	Conc. (Bio-endpoints)	Association (Ox-capacity vs Bio-endpoints)	Reference
PM_{10}, $PM_{2.5}$ and UFP sampled at 8 locations (24 samples)	DTT	-	*In vitro* cytotoxicity (MTT test) and induction of TNF-α, IL-6 and MIP-2 in RAW264.7 cells	6.25-100 µg/mL	Statistical significant correlation with cytotoxicity (β = -145, P < 0,0001)<br>*Note: Significant correlation with cytokines obtained after excluding the sample with the highest oxidative capacity (β = 45/80/647, P < 0,0001)*	Steenhof et al. 2011
Wood smoke particles and fine PM (4 samples)	DCFH<br>ESR with DMPO	1.56-50 µg/mL<br>1 mg/mL	*In vitro* intracellular ROS, DNA damage (comet assay with and without FPG, 8-oxodG, εdA, εdG, and DNA adducts), and induction of MCP-1, TNF-α, LFA-1, CXCL8, HO-1, OGG1 in THP-1 and A549 cells, *in vivo* inflammation, oxidative stress and DNA damage in rat lung and liver	*In vitro*: 1.56-100 µg/mL<br>*In vivo*: 0.64 mg/kg (~128 µg/rat)	No apparent association<br>*Note: the particle with the highest oxidative capacity also induced the highest level of HO-1 expression in THP-1 cells*	Danielsen et al. 2010, and 2011*
PM from 2 locations sampled at two different time points (4 samples)	ESR with H_2O_2 and DMPO	-	*In vitro* RBC-hemolysis	80 µg/mL	No apparent association	Quintana et al. 2011
Diesel and biodiesel exhaust particles (8 samples)	DTT<br>Depletion of AA	10 µg/mL<br>12 µg/mL	*In vitro* cytotoxicity and IL-6 release in BEAS-2B cells	3.125-200 µg/mL	No apparent association	Gerlofs-Nijland et al. 2013
Day-to-day variations in $PM_{2.5}$ (10 days)	DTT	-	*In vitro* ROS formation in primary rat AM	PM2.5: 3-87 µg/m³	Statistical significant correlation (r_s = 0.86)<br>*Note: OP^{DTT} also strongly correlated with organic carbon (r_s = 0.80) and water-soluble organic carbon (r_s = 0.90)*	Delfino et al. 2013
PM_{10} (6 samples)	DTT	-	*In vitro* intracellular ROS and TNF-α and CXCL8 release in J774A.1 and A549 cells	ROS: 100-400 µg/mL<br>Cytokines: 25-100 µg/mL	No statistical significant correlation	Lu et al. 2014
Welding fumes (3 samples)	ESR with H_2O_2 and DMPO		Cytotoxicity (mitochondrial dysfunction) intracellular ROS (ESR w/ DMPO), DNA damage (Comet assay), phagocytosis, cytokine release (TNF-α, IL-6, IL-1β) in RAW264.7 cells	50 and 250 µg/mL	No apparent association	Badding et al. 2014
$PM_{2.5}$ sampled over 2 months at 2 sites (54 samples)	DTT	-<br>Section of PM-filter (different conc. for each sample)	*In vitro* induction of TNF-α, and IL-6 release in A549 cells (24 and 48 h)	-<br>Aqueous extracts of PM-filter sections (corresponding to 0.5-2.5 mg/mL ?)	Statistically significant correlation (TNF-α: R^2= 0.65-0.74, p < 0.001 ; IL-6: R^2= 0.80-0.91, p < 0.001)<br>*Note: Exposure concentrations not clearly specified but appears extremely high*	Liu et al. 2014

Studies marked in green show significant correlation/association between at least one OP-measure and all biological effects assessed, confirmed by statistical analysis. Studies marked in blue show significant correlation/association between at least one OP-measure and one biological effect confirmed by statistical analysis. Studies marked in grey show possible correlations/associations, but lacks confirmation by statistical analysis. Studies marked in red show no significant correlation/association between any OP-measures and any biological effects, confirmed by statistical analysis. Studies marked in white show no apparent correlations/associations between any OP-measures and any biological effects, but lacks confirmation by statistical analysis. * The two studies by Danielsen et al (2010;2011) were counted as one, as they were based on the same particles and measurements of oxidative capacity.

Table S1 (continued). Overview of cell culture and animal studies where biological effects of PM and combustion particles have been examined and compared with acellular oxidative capacity

Particles	Ox-capacity (Cell-free)	Conc. (OP)	Biological endpoints	Conc. (Bio-endpoints)	Association (Ox-capacity vs Bio-endpoints)	Reference
Water-soluble fractions of PM of different size, from separate sites and seasons (20 samples)	DDT	- Not clearly stated (different conc. for each sample)	*In vitro* Cytotoxicity (MTT and LDH) and DNA damage (Comet assay)	- Not clearly stated (different conc. for each sample)	No apparent overall association when considering all PM samples together.<br><br>However, moderate but statistically significant correlation between OP^{DTT} and MTT when PM sampled at different sites and seasons were analyzed separately (R^2 = 0.30-0.46), and strong correlation between OP^{DTT} and LDH in the winter samples (R^2 = 0.78-0.79). Implies that other factors contribute.	Velali et al. 2015
8 diesel exhaust particles	DTT	-	*In vitro* cytotoxicity in SVEC4-10 cells: cell proliferation (colony formation), MTT test and wound healing (scratch test)	5-100 µg/mL	No apparent association<br><br>(2-fold variation in OP, but no variation in cytotoxicity)	Fox et al. 2015
108 PM samples	ESR with H_2O_2 and DMPO	-	*In vitro* cytotoxicity (Neutral red uptake), cytokines (IL-8) and DNA damage (Comet assay with FPG) in BEAS-2B cells	12.5-100 µg/mL	No statistical significant correlation	Van Den Heuvel et al 2016
4 diesel exhaust particles	DTT assay	-	*In vitro* gene expression of TNF-α and HO-1 in Raw 264.7 cells	-	Statistically significant correlation with both endpoints	Karavalakis et al. 2017
9 $PM_{2.5}$ and 1 diesel exhaust particle	DTT assay, depletion of GSH and AA, plasmid scission assay	10-100 µg/mL	*In vitro* intracellular ROS (DCFH), gene expression of antioxidants (SOD and HO-1), cytokines (IL-6) and metabolizing enzymes (CYP1A1) in NCH-H292 cells	1-10 µg/mL	Statistically significant correlation<br><br>*Note: OP^{AA} and OP^{GSH} strongest correlated with all endpoints. OP^{GSH} and OP^{DTT} not correlated with IL-6.*	Crobeddu et al. 2017
95 PM_{10} samples	ESR with H_2O_2 and DMPO	-	*In vitro* cytotoxicity (Neutral red uptake), and cytokines (IL-8) in BEAS-2B cells. Bacterial mutagenicity by Ames test.	12.5-100 µg/mL	No statistical significant correlation with IL-8 or cytotoxicity<br><br>Statistically significant correlation between OP and bacterial mutagenicity.	Van Den Heuvel et al. 2018
$PM_{0.25}$ from two airports	ESR with H_2O_2 and DMPO, depletion of GSH and AA	50 µg/mL<br><br>12.5 µg/mL	*In vitro* cytotoxicity (MTT), intracellular ROS (H_2DCFDA), and cytokine release (TNF-α, IL-6 and IL-8) in 16HBE cells.	10 and 100 µg/mL	Statistical significant correlation between OP and intracellular ROS<br><br>No apparent association between OP and cytotoxicity or proinflammatory cytokines	He et al. 20018
3 diesel particles	DTT (PM ethanol extracts)	-	*IN vitro* gene expression of metabolizing enzymes (CYP1A1 and -1B1), cytokine release (TNF-α and IL-8), intracellular Ca^{2+}.	CYP1/cytokines: 200 µg/mL (50 µg/cm²)<br><br>Ca^{2+}: 2.3 mg/ml	Statistical significant correlation between OP and CYP1 expression (R^2>0.9), but low correlation with cytokines (R^2=0.2 and 0.26)	Jaramillo et al. 2018

Studies marked in green show significant correlation/association between at least one OP-measure and all biological effects assessed, confirmed by statistical analysis. Studies marked in blue show significant correlation/association between at least one OP-measure and one biological effect confirmed by statistical analysis. Studies marked in grey show possible correlations/associations, but lacks confirmation by statistical analysis. Studies marked in red show no significant correlation/association between any OP-measures and any biological effects, confirmed by statistical analysis. Studies marked in white show no apparent correlations/associations between any OP-measures and any biological effects, but lacks confirmation by statistical analysis.

Table S2. Overview of epidemiological and human exposure studies where health effects of PM and combustion particles have been examined and compared with acellular oxidative capacity

Particles	OP (Cell-free)	Number of samples	Biological endpoints	Conc. (Bio-endpoints)	Association (Ox-capacity vs Bio-endpoints)	Reference
Utah valley PM_{10} collected different times (3 samples)	Deoxyribose assay	3	Pulmonary inflammation in human volunteers (total cells, PMNs, total protein, albumin, fibronectin, α 1-antitrypsin, tissue factor, fibrinogen, IL-1β, TNF-α, and CXCL8 in BALF)	100 and 500 µg/person	Possible association with all endpoints<br><br>*Note: effects may be due to high zinc levels which co-variated with redox active metals*	Ghio and Devlin 2001
Metal-rich and metal-poor $PM_{2.5}$ (2 samples)	ESR with H_2O_2 and DMPO	2	Pulmonary inflammation in **human volunteers** (total cells, PMNs, monocytes, IL-6, IL-8. TNF-α in BALF and ROS generation by BAL-cells)	100 µg/person	Possible association with all endpoints<br><br>*Note: Effects were also associated with the most abundant metal, zinc*	Schaumann et al. 2004
TSP, PM_{10} and $PM_{2.5}$ sampled at 6 schools sampled at 4 days	ESR with ascorbate and DMPO	-	Lung function (FEV_1, FVC, $FEF_{50\%}$) in 651 children (8-13 years) attending the same 6 schools	Outdoor air PM conc.:<br><br>TSP:<br>59-101 µg/m³<br>PM_{10}:<br>28-59 µg/m³<br>$PM_{2.5}$:<br>15-24 µg/m³	Statistically significant negative association with lung function ($P < 0.05$)	Hogervorst et al. 2006
Predicted weekly PM_{10} mass and oxidative potential, 2002-2006	Depletion of GSH in synthetic lung lining fluid	Modeled based on 34 monitoring sites and 841 measures	Carotide intima-media thickness in cohort of 2348 (mean age: 61 yr)	25.0 ± 0.6 µg/m³ (mean ± SD)	PM_{10} mass more strongly correlated than PM_{10} OP	Tonne et al. 2012
Day-to-day variations in $PM_{2.5}$ (10 days)	DTT	10	Airway inflammation (exhaled NO) in 45 children with asthma (9-18 yr)	3-87 µg/m³	Positive association with exhaled NO in children	Delfino et al. 2013
PM_{10} levels measured on day of admission and 14 days before/after	Antioxidant depletion (GSH, AA, uric acid) in synthetic lung lining fluid	-	160 asthma/COPD exacerbations in 151 patients<br>(bi-directional case-crossover study)	-	No statistical significant correlation with asthma/COPD admissions<br><br>*Note: The same authors had earlier reported an association with PM_{10} mass*	Canova et al. 2014
$PM_{2.5}$ sampled from June 2012 to April 2018	DTT assay on water-soluble $PM_{2.5}$ extracts	227 samples.<br><br>DTT activity modelled for 1998-2009	Emergency department visits for asthma/wheezing and congestive heart failure	-	OP more strongly associated than $PM_{2.5}$ mass	Bates et al. 2015
The RAPTES-project<br><br>Real life PM-exposure at 5 locations	Depletion of GSH and AA	-	Airway inflammation (exhaled NO), lung function (FEV_1, FVC), nasal inflammation (IL-6, IL-8, and lactoferrin), and various vascular inflammatory and coagulative markers in 31 human volunteers	5 h exposure<br><br>PM_{10}:<br>26-394 µg/m³<br>$PM_{2.5}$:<br>16-140 µg/m³<br>$PM_{10-2.5}$:<br>9-252 µg/m³<br>PNC:<br>9-67 x10³/cm³	No apparent association between acellular oxidative capacity of the different PM-fractions sampled at each location and any of the assessed endpoints	Janssen et al. 2015; Steenhof et al. 2013 and 2014; Strak et al. 2012, 2013a, and 2013b*

Studies marked in green show significant correlation/association between at least one OP-measure and all biological effects assessed, confirmed by statistical analysis. Studies marked in grey show possible correlations/associations, but lacks confirmation by statistical analysis. Studies marked in red show no significant correlation/association between any OP-measures and any biological effects, confirmed by statistical analysis. Studies marked in white show no apparent correlations/associations between any OP-measures and any biological effects, but lacks confirmation by statistical analysis. *The 6 publications under the RAPTES-project were apparently based on analysis from the same individuals and exposures, and have been considered as one study.

Table S2 (continued). Overview of epidemiological and human exposure studies where health effects of PM and combustion particles have been examined and compared with acellular oxidative capacity

Particles	OP (Cell-free)	Number of samples	Biological endpoints	Conc. (Bio-endpoints)	Association (Ox-capacity vs Bio-endpoints)	Reference
PM_{10} and $PM_{2.5}$, central London, UK	Depletion of GSH and AA in synthetic lung lining fluid	Daily OP levels for 685/703 days in 2011-2012	All-cause non-accidental mortality, death from cardiovascular and respiratory disease, stratified by age (0-14, 15-64 and 65+ years)	PM10: 9-32 µg/m^3 PM2.5: 5-25 µg/m^3 (10th-90th percentile)	No statistical significant association with OP in complete population. PM_{10} OP^{AA} showed stronger negatively association with respiratory mortality in elderly (65+), than PM_{10} and $PM_{2.5}$ mass.	Atkinson et al. 2016
$PM_{2.5}$, Netherlands and Beligium	DTT assay, ESR with H_2O_2 and DMPO	Measures of $PM^{2.5}$ OP from 40 sites, applied to land-use regression (LUR) model	Doctor diagnosed asthma, prevalence of asthma symptoms, hay fever, and rhinitis by age 14. Allergic sensitization, lung function (FEV_1, FEVC, FEF_{25-75}) and FeNO at age 12, in a birth cohort of 3701 children	-	OP^{DTT}, but not OP^{ESR}, statistically significantly associated with asthma incident and symptoms, rhinitis, and lung function.<br><br>*Note: Most associations with lung function, but not symptoms, were lost when adjusting for NO_2.*	Yang et al. 2016
$PM_{2.5}$, Atlanta, USA	DTT assay	OP of water-soluble fraction of 196 daily samples (2011-2012), used to model OP for 1998-2009	Hospital visits for respiratory (pneumonia, COPD, asthma/wheeze) and cardiovascular disease (ischemic heart disease, congestive heart failure) in Atlanta 1998-2009	-	Modelled OP^{DTT} were statistically significantly associated with asthma,/wheeze and IHD<br><br>Note: association with $PM_{2.5}$ not assessed, but OP^{DTT} co-varied strongly with $PM_{2.5}$ mass (r = 0.49-0.86)	Abrams et al. 2017
$PM_{2.5}$, southeastern USA	DTT assay, depletion of AA	OP of water-soluble fraction of 500 samples, used to model OP for 1998-2009	Hospital visits for respiratory (pneumonia, COPD, asthma/wheeze) and cardiovascular disease (ischemic heart disease, congestive heart failure) in Atlanta 1998-2009	-	Modelled OP^{DTT}, but not OP^{AA}, were statistically significantly associated with asthma,/wheeze and CHF<br><br>Note: association with $PM_{2.5}$ not assessed, but OP^{DTT} co-varied strongly with $PM_{2.5}$ mass (r = 0.49-0.86)	Fang et al. 2016
$PM_{10-2.5}$, $PM_{2.5-0.18}$, $PM_{0.18}$ and black carbon, Los Angeles, USA	DTT assay	Five days of PM sampling (coarse, fine, ultrafine) prior to each clinical visit in study.	Microvascular function (reactive hyperemia index: RHI) in a cohort panel study of 93 non-smoking adults (65-96 years old)	-	$PM_{0.18}$ OP^{DTT}, but not $PM_{2.5-0.18}$ OP^{DTT} or $PM_{10-2.5}$ OP^{DTT}, was statistically significantly associated with reduced RHI.	Zhang et al. 2016
PM2.5, Montreal, Canada	Depletion of GSH and AA in synthetic lung lining fluid	Personal exposure of 62 asthmatic children of 10 days	Airway inflammation (FeNO)	-	OP^{GSH}, but not OP^{AA} or $PM_{2.5}$ mass, was statistically significantly associated with FeNO	Maikawa et al. 2016
PM2.5, Ontario, Canada	Depletion of GSH and AA in synthetic lung lining fluid	PM2.5 sampled in 30 cities in Ontario	Cause-specific mortality (all non-accidental, lung cancer, cardio-metabolic, ischemic heart disease, respiratory disease)	-	OP^{GSH} more strongly associated with lung cancer deaths than $PM_{2.5}$ mass, while $PM_{2.5}$ mass showed stronger association with all non-specific mortality.<br><br>*No associations were observed with OP^{AA}.*	Weichenthal et al. 2016A

Studies marked in green show significant correlation/association between at least one OP-measure and all biological effects assessed, confirmed by statistical analysis. Studies marked in blue show significant correlation/association between at least one OP-measure and one biological effect confirmed by statistical analysis. Studies marked in red show no significant correlation/association between any OP-measures and any biological effects, confirmed by statistical analysis.

Table S2 (continued). Overview of epidemiological and human exposure studies where health effects of PM and combustion particles have been examined and compared with acellular oxidative capacity

**Particles**	**OP** (Cell-free)	**Number of samples**	**Biological endpoints**	**Conc.** (Bio-endpoints)	**Association** (Ox-capacity vs Bio-endpoints)	**Reference**
PM2.5, Ontario, Canada	Depletion of GSH and AA in synthetic lung lining fluid	PM2.5 sampled in 16-31 cities in Ontario	Emergency room visits for respiratory illness and myocardial infarctions, and birth outcomes (preterm birth and low birth weight).	-	OP^{GSH} modified association between $PM_{2.5}$ respiratory illness, myocardial infarctions and birth outcomes (stronger increases in $PM_{2.5}$-associated risks were observed in regions with high OP^{GSH}). *No effect-modifications were observed with OP^{AA}.*	Weichenthal et al. 2016B, 2016C, Lavigne et al. 2018**
PM_{10}, $PM_{2.5}$ and $PM_{10-2.5}$, Netherlands	DTT assay, ESR with H_2O_2 and DMPO	Measures of $PM_{2.5}$ OP from 40 sites, applied to land-use regression (LUR) model	Diabetes prevalence among 289.703 adults	-	OP^{DTT}, more strongly associated with diabetes than PM-mass. NO association with OP^{ESR}. *Note: strong associations also observed for NO_2*	Strak et al. 2017
$PM_{10-2.5}$, $PM_{2.5-0.15}$ and $PM_{0.3}$ Ontario, Canada	Depletion of GSH and AA in synthetic lung lining fluid	Controlled exposures to CAPs (3x53)	Clinical trial with 53 healthy volunteers (mean age 28) in chamber<br><br>Blood biomarkers: ET1, IL-6, CRP, VEGF, MDA, S100, NSE, UCHL1, cortisol, BDNF<br><br>Urinary biomarkers: VEGF, 8-OHDG, MDA, VMA, HVA, cortisol	$PM_{10-2.5}$ (µg/m^3): 212.6 ± 51.8<br><br>$PM_{2.5-0.15}$ (µg/m^3): 238.4 ± 62.0<br><br>$PM_{0.3}$(µg/m^3): 120.0 ± 72.0	OP^{GSH} was statistically significantly associated with blood IL-6, VEGF and S100, and urinary 8-OHDG. OP^{AA} was associated with blood UCHL1, and urinary MDA. $PM_{2.5}$ mass was associated with blood MDA and urinary 8-OHDG.<br><br>*Note: as several GSH measurements were below detection levels, OPGSH associations assessed based on a binary model (above or below detection limit)*	Liu et al. 2018

Studies marked in green show significant correlation/association between at least one OP-measure and all biological effects assessed, confirmed by statistical analysis. Studies marked in blue show significant correlation/association between at least one OP-measure and one biological effect confirmed by statistical analysis. **The three publications from Ontario, by Weichenthal and colleagues were counted as one, as these were based on the same exposures (PM mass and OP metrics from same sites and sample periods).

Fig S1

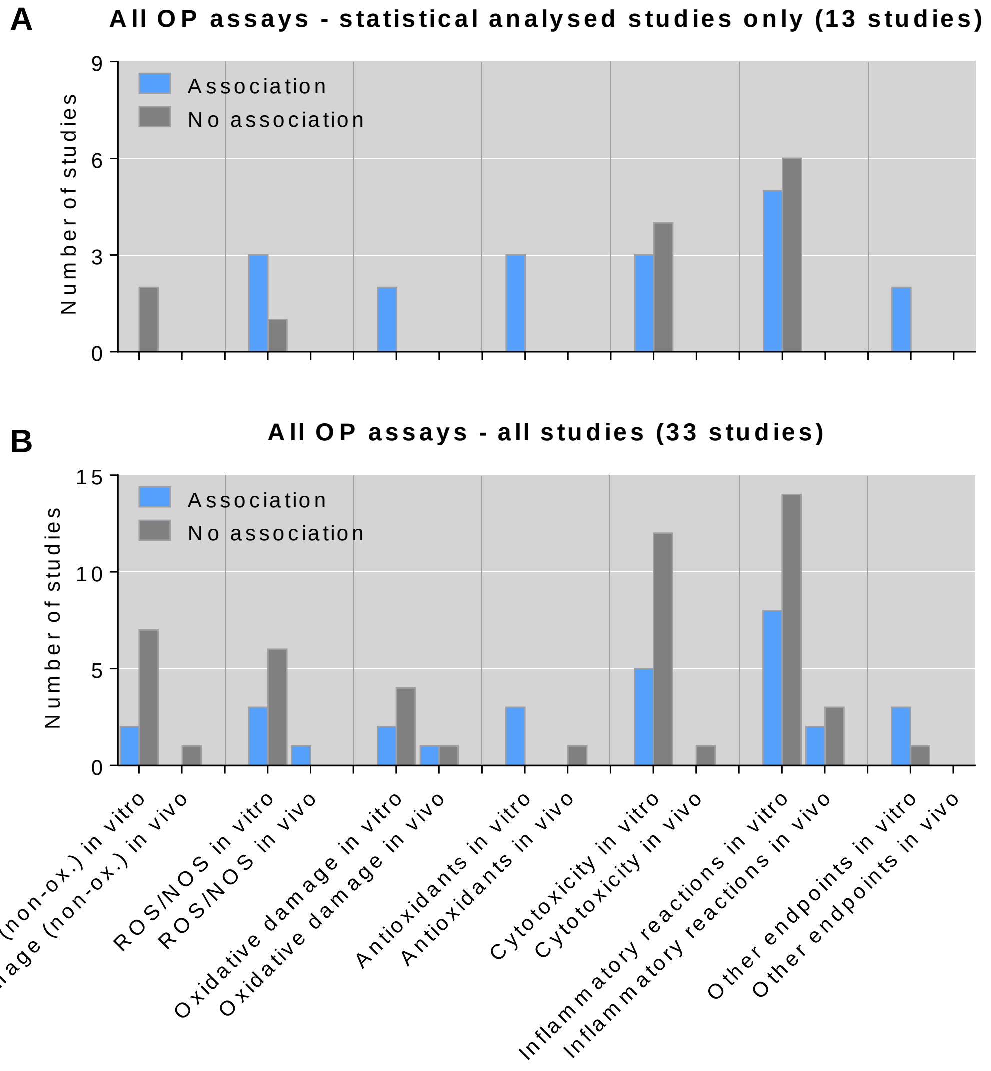

Figure S1. Possible association between all OP assays (pooled) and biological effects of PM in experimental studies *in vitro* and *in vivo*. The figure displays the number of studies showing an association or no association between OP measured by any assay specific biological effects *in vitro* (cell cultures), by statistical analysis only (A) or also including studies were statistics were not applied (B). Data in B was based on comparing rank order of OP vs rank order og biological effects of different PM samples. “Oxidative damage” include lipid peroxidation and oxidative DNA damage. “Other endpoints” include cellular signaling, proliferation. As some studies have explored association between OP and several different biological effects, the sum of the individual columns exceeds the total number of studies given in the figure title.

Fig S2

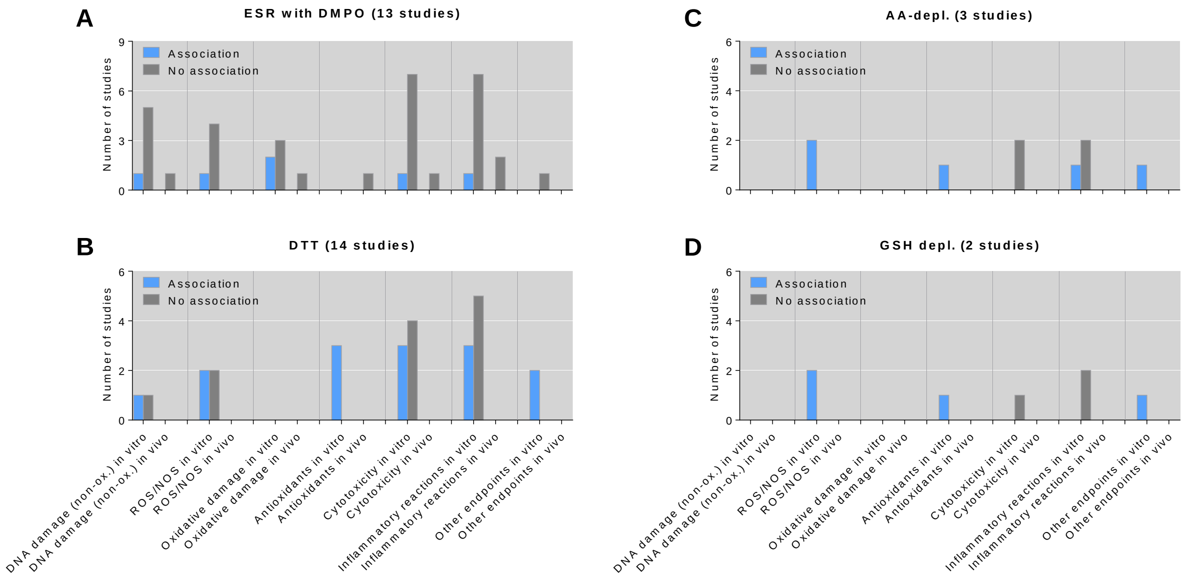

Figure S2. Possible association between specific OP assays and biological effects of PM in experimental studies *in vitro*. The figure displays the number of studies showing an apparent association or no apparent association between OP measured by ESR with DMPO as spin trap (A), DTT-assay (B), AA-depletion (C) or GSH-depletion (D) and specific biological effects *in vitro* (cell cultures). The data includes not only results from studies applying statistical analysis of the association between OP and effects, but also studies were statistical analysis were not applied. When statistical analysis were not available, the rank order of OP vs rank order of biological effects of different PM samples were compared. “Oxidative damage” include lipid peroxidation and oxidative DNA damage. “Other endpoints” include cellular signaling, proliferation. As some studies have explored association between OP and several different biological effects, the sum of the individual columns exceeds the total number of studies given in the figure title. *AA – ascorbic acid; DMPO - 5,5-dimethyl-pyrroline N-oxide; DTT – dithiothreitol; ESR – electron spin resonance; GSH – reduced glutathione.*

References

Abrams, J. Y., R. J. Weber, M. Klein, S. E. Sarnat, H. H. Chang, M. J. Strickland, V. Verma, T. Fang, J. T. Bates, J. A. Mulholland, A. G. Russell, and P. E. Tolbert. 2017. 'Associations between Ambient Fine Particulate Oxidative Potential and Cardiorespiratory Emergency Department Visits', *Environ Health Perspect*, 125: 107008.

Akhtar, U.S., R.D. McWhinney, N. Rastogi, J.P. Abbatt, G.J. Evans, and J.A. Scott. 2010. 'Cytotoxic and proinflammatory effects of ambient and source-related particulate matter (PM) in relation to the production of reactive oxygen species (ROS) and cytokine adsorption by particles', *Inhal. Toxicol*, 22 Suppl 2: 37-47.

Atkinson, R. W., E. Samoli, A. Analitis, G. W. Fuller, D. C. Green, H. R. Anderson, E. Purdie, C. Dunster, L. Aitlhadj, F. J. Kelly, and I. S. Mudway. 2016. 'Short-term associations between particle oxidative potential and daily mortality and hospital admissions in London', *Int J Hyg Environ Health*, 219: 566-72.

Badding, M.A., N.R. Fix, J.M. Antonini, and S.S. Leonard. 2014. 'A comparison of cytotoxicity and oxidative stress from welding fumes generated with a new nickel-, copper-based consumable versus mild and stainless steel-based welding in RAW 264.7 mouse macrophages', *PLoS. One*, 9: e101310.

Bates, J.T., R.J. Weber, J. Abrams, V. Verma, T. Fang, M. Klein, M.J. Strickland, S.E. Sarnat, H.H. Chang, J.A. Mulholland, P.E. Tolbert, and A.G. Russell. 2015. 'Reactive Oxygen Species Generation Linked to Sources of Atmospheric Particulate Matter and Cardiorespiratory Effects', *Environ. Sci. Technol*, 49: 13605-12.

Beck-Speier, I., N. Dayal, E. Karg, K.L. Maier, G. Schumann, H. Schulz, M. Semmler, S. Takenaka, K. Stettmaier, W. Bors, A. Ghio, J.M. Samet, and J. Heyder. 2005. 'Oxidative stress and lipid mediators induced in alveolar macrophages by ultrafine particles', *Free. Radic. Biol. Med*, 38: 1080-92.

Canova, C., C. Minelli, C. Dunster, F. Kelly, P.L. Shah, C. Caneja, M.K. Tumilty, and P. Burney. 2014. 'PM10 oxidative properties and asthma and COPD', *Epidemiology*, 25: 467-68.

Crobeddu, B., L. Aragao-Santiago, L. C. Bui, S. Boland, and A. Baeza Squiban. 2017. 'Oxidative potential of particulate matter 2.5 as predictive indicator of cellular stress', *Environ Pollut*, 230: 125-33.

Danielsen, P.H., S. Loft, N.R. Jacobsen, K.A. Jensen, H. Autrup, J.L. Ravanat, H. Wallin, and P. Moller. 2010. 'Oxidative stress, inflammation, and DNA damage in rats after intratracheal instillation or oral exposure to ambient air and wood smoke particulate matter', *Toxicol. Sci*, 118: 574-85.

Danielsen, P.H., P. Moller, K.A. Jensen, A.K. Sharma, H. Wallin, R. Bossi, H. Autrup, L. Molhave, J.L. Ravanat, J.J. Briede, T.M. de Kok, and S. Loft. 2011. 'Oxidative stress, DNA damage, and inflammation induced by ambient air and wood smoke particulate matter in human A549 and THP-1 cell lines', *Chem. Res. Toxicol*, 24: 168-84.

De Vizcaya-Ruiz, A., O. Gutierrez-CastiBarbier, J.L. Usachenko, M. Uribe-Ramirez, M.E. Cebrian, V. Mugica-Alvarez, J. Sepulveda, I. Rosas, E. Salinas, C. Garcia-Cuellar, F. Martinez, E. Alfaro-Moreno, V. Torres-Flores, A. Osornio-Vargas, C. Sioutas, P.M. Fine, M. Singh, M.D. Geller, T. Kuhn, A.H Miguel, A. Eiguren-Fernandez, R.H. Schiestl, R. Reliene, and J. Froines. 2006. 'Characterization and in vitro biological effects of concentrated particulate matter from Mexico City', *Atmospheric Environ*, 40: S583-S92.

Delfino, R.J., N. Staimer, T. Tjoa, D.L. Gillen, J.J. Schauer, and M.M. Shafer. 2013. 'Airway inflammation and oxidative potential of air pollutant particles in a pediatric asthma panel', *J Expo Sci Environ Epidemiol*, 23: 466-73.

Dellinger, B., W.A. Pryor, R. Cueto, G.L. Squadrito, V. Hegde, and W.A. Deutsch. 2001. 'Role of free radicals in the toxicity of airborne fine particulate matter', *Chem Res Toxicol*, 14: 1371-77.

Fang, T., V. Verma, J. T. Bates, J. Abrams, M. Klein, M. J. Strickland, S. E. Sarnat, H. H. Chang, J. A. Mulholland, P. E. Tolbert, A. G. Russell, and R. J. Weber. 2016. 'Oxidative potential of ambient water-soluble PM2.5 in the southeastern United States: contrasts in sources and health

associations between ascorbic acid (AA) and dithiothreitol (DTT) assays', *Atmos. Chem. Phys.*, 16: 3865-79.
Fox, J.R., D.P. Cox, B.E. Drury, T.R. Gould, T.J. Kavanagh, M.H. Paulsen, L. Sheppard, C.D. Simpson, J.A. Stewart, T.V. Larson, and J.D. Kaufman. 2015. 'Chemical characterization and in vitro toxicity of diesel exhaust particulate matter generated under varying conditions', *Air Qual. Atmos. Health*, 8: 507-19.
Frampton, M.W., A.J. Ghio, J.M. Samet, J.L. Carson, J.D. Carter, and R.B. Devlin. 1999. 'Effects of aqueous extracts of PM(10) filters from the Utah valley on human airway epithelial cells', *Am. J. Physiol*, 277: L960-L67.
Gerlofs-Nijland, M.E., A.I. Totlandsdal, T. Tzamkiozis, D.L. Leseman, Z. Samaras, M. Lag, P. Schwarze, L. Ntziachristos, and F.R. Cassee. 2013. 'Cell toxicity and oxidative potential of engine exhaust particles: impact of using particulate filter or biodiesel fuel blend', *Environ Sci Technol*, 47: 5931-38.
Ghio, A.J., and R.B. Devlin. 2001. 'Inflammatory lung injury after bronchial instillation of air pollution particles', *Am. J. Respir. Crit Care Med*, 164: 704-08.
Ghio, A.J., J. Stonehuerner, L.A. Dailey, and J.D. Carter. 1999. 'Metals associated with both the water-soluble and insoluble fractions of an ambient air pollution particle catalyze an oxidative stress', *Inhal. Toxicol*, 11: 37-49.
He, R. W., F. Shirmohammadi, M. E. Gerlofs-Nijland, C. Sioutas, and F. R. Cassee. 2018. 'Pro-inflammatory responses to PM0.25 from airport and urban traffic emissions', *Sci Total Environ*, 640-641: 997-1003.
Hogervorst, J.G., T.M. de Kok, J.J. Briede, G. Wesseling, J.C. Kleinjans, and C.P. van Schayck. 2006. 'Relationship between radical generation by urban ambient particulate matter and pulmonary function of school children', *J. Toxicol. Environ. Health A*, 69: 245-62.
Janssen, N.A., M. Strak, A. Yang, B. Hellack, F.J. Kelly, T.A. Kuhlbusch, R.M. Harrison, B. Brunekreef, F.R. Cassee, M. Steenhof, and G. Hoek. 2015. 'Associations between three specific a-cellular measures of the oxidative potential of particulate matter and markers of acute airway and nasal inflammation in healthy volunteers', *Occup Environ Med*, 72: 49-56.
Jaramillo, I. C., A. Sturrock, H. Ghiassi, D. J. Woller, C. E. Deering-Rice, J. S. Lighty, R. Paine, C. Reilly, and K. E. Kelly. 2018. 'Effects of fuel components and combustion particle physicochemical properties on toxicological responses of lung cells', *J Environ Sci Health A Tox Hazard Subst Environ Eng*, 53: 295-309.
Karavalakis, G., N. Gysel, D. A. Schmitz, A. K. Cho, C. Sioutas, J. J. Schauer, D. R. Cocker, and T. D. Durbin. 2017. 'Impact of biodiesel on regulated and unregulated emissions, and redox and proinflammatory properties of PM emitted from heavy-duty vehicles', *Sci Total Environ*, 584-585: 1230-38.
Lavigne, E., R. T. Burnett, D. M. Stieb, G. J. Evans, K. J. Godri Pollitt, H. Chen, D. van Rijswijk, and S. Weichenthal. 2018. 'Fine Particulate Air Pollution and Adverse Birth Outcomes: Effect Modification by Regional Nonvolatile Oxidative Potential', *Environ Health Perspect*, 126: 077012.
Leonard, S.S., V. Castranova, B.T. Chen, D. Schwegler-Berry, M. Hoover, C. Piacitelli, and D.M. Gaughan. 2007. 'Particle size-dependent radical generation from wildland fire smoke', *Toxicology*, 236: 103-13.
Li, N., C. Sioutas, A. Cho, D. Schmitz, C. Misra, J. Sempf, M. Wang, T. Oberley, J. Froines, and A. Nel. 2003. 'Ultrafine particulate pollutants induce oxidative stress and mitochondrial damage', *Environ Health Perspect*, 111: 455-60.
Liu, L., B. Urch, M. Szyszkowicz, G. Evans, M. Speck, A. Van Huang, K. Leingartner, R. H. Shutt, G. Pelletier, D. R. Gold, J. R. Brook, K. Godri Pollitt, and F. S. Silverman. 2018. 'Metals and oxidative potential in urban particulate matter influence systemic inflammatory and neural biomarkers: A controlled exposure study', *Environ Int*, 121: 1331-40.

Liu, Q., J. Baumgartner, Y. Zhang, Y. Liu, Y. Sun, and M. Zhang. 2014. 'Oxidative potential and inflammatory impacts of source apportioned ambient air pollution in Beijing', *Environ. Sci. Technol*, 48: 12920-29.

Lu, Y., S. Su, W. Jin, B. Wang, N. Li, H. Shen, W. Li, Y. Huang, H. Chen, Y. Zhang, Y. Chen, N. Lin, X. Wang, and S. Tao. 2014. 'Characteristics and cellular effects of ambient particulate matter from Beijing', *Environ Pollut*, 191: 63-69.

Maikawa, C. L., S. Weichenthal, A. J. Wheeler, N. A. Dobbin, A. Smargiassi, G. Evans, L. Liu, M. S. Goldberg, and K. J. Pollitt. 2016. 'Particulate Oxidative Burden as a Predictor of Exhaled Nitric Oxide in Children with Asthma', *Environ Health Perspect*, 124: 1616-22.

Molinelli, A.R., M.C. Madden, J.K. McGee, J.G. Stonehuerner, and A.J. Ghio. 2002. 'Effect of metal removal on the toxicity of airborne particulate matter from the Utah Valley', *Inhal Toxicol*, 14: 1069-86.

Quintana, R., J. Serrano, V. Gomez, Foy B. de, J. Miranda, C. Garcia-Cuellar, E. Vega, I. Vazquez-Lopez, L.T. Molina, N. Manzano-Leon, I. Rosas, and A.R. Osornio-Vargas. 2011. 'The oxidative potential and biological effects induced by PM10 obtained in Mexico City and at a receptor site during the MILAGRO Campaign', *Environ. Pollut*, 159: 3446-54.

Repine, J.E., O.K. Reiss, N. Elkins, A.R. Chughtai, and D.M. Smith. 2008. 'Effects of fine carbonaceous particles containing high and low unpaired electron spin densities on lungs of female mice', *Transl. Res*, 152: 185-93.

Salonen, R.O., A.I. Halinen, A.S. Pennanen, M.R. Hirvonen, M. Sillanpaa, R. Hillamo, T. Shi, P. Borm, E. Sandell, T. Koskentalo, and P. Aarnio. 2004. 'Chemical and in vitro toxicologic characterization of wintertime and springtime urban-air particles with an aerodynamic diameter below 10 microm in Helsinki', *Scand. J. Work Environ. Health*, 30 Suppl 2: 80-90.

Schaumann, F., P.J. Borm, A. Herbrich, J. Knoch, M. Pitz, R.P. Schins, B. Luettig, J.M. Hohlfeld, J. Heinrich, and N. Krug. 2004. 'Metal-rich ambient particles (particulate matter 2.5) cause airway inflammation in healthy subjects', *Am. J. Respir. Crit Care Med*, 170: 898-903.

Schins, R.P., J.H. Lightbody, P.J. Borm, T. Shi, K. Donaldson, and V. Stone. 2004. 'Inflammatory effects of coarse and fine particulate matter in relation to chemical and biological constituents', *Toxicol. Appl. Pharmacol*, 195: 1-11.

Shi, T., R. Duffin, P.J. Borm, H. Li, C. Weishaupt, and R.P. Schins. 2006. 'Hydroxyl-radical-dependent DNA damage by ambient particulate matter from contrasting sampling locations', *Environ Res*, 101: 18-24.

Shi, T., A.M. Knaapen, J. Begerow, W. Birmili, P.J. Borm, and R.P. Schins. 2003. 'Temporal variation of hydroxyl radical generation and 8-hydroxy-2'-deoxyguanosine formation by coarse and fine particulate matter', *Occup. Environ. Med*, 60: 315-21.

Steenhof, M., I. Gosens, M. Strak, K.J. Godri, G. Hoek, F.R. Cassee, I.S. Mudway, F.J. Kelly, R.M. Harrison, E. Lebret, B. Brunekreef, N.A. Janssen, and R.H. Pieters. 2011. 'In vitro toxicity of particulate matter (PM) collected at different sites in the Netherlands is associated with PM composition, size fraction and oxidative potential--the RAPTES project', *Part Fibre Toxicol*, 8: 26.

Steenhof, M., N.A. Janssen, M. Strak, G. Hoek, I. Gosens, I.S. Mudway, F.J. Kelly, R.M. Harrison, R.H. Pieters, F.R. Cassee, and B. Brunekreef. 2014. 'Air pollution exposure affects circulating white blood cell counts in healthy subjects: the role of particle composition, oxidative potential and gaseous pollutants - the RAPTES project', *Inhal Toxicol*, 26: 141-65.

Steenhof, M., I.S. Mudway, I. Gosens, G. Hoek, K.J. Godri, F.J. Kelly, R.M. Harrison, R.H. Pieters, F.R. Cassee, E. Lebret, B.A. Brunekreef, M. Strak, and N.A. Janssen. 2013. 'Acute nasal pro-inflammatory response to air pollution depends on characteristics other than particle mass concentration or oxidative potential: the RAPTES project', *Occup Environ Med*, 70: 341-48.

Strak, M., G. Hoek, K.J. Godri, I. Gosens, I.S. Mudway, Oerle R. van, H.M. Spronk, F.R. Cassee, E. Lebret, F.J. Kelly, R.M. Harrison, B. Brunekreef, M. Steenhof, and N.A. Janssen. 2013. 'Composition of PM affects acute vascular inflammatory and coagulative markers - the RAPTES project', *PLoS One*, 8: e58944.

Strak, M., G. Hoek, M. Steenhof, E. Kilinc, K.J. Godri, I. Gosens, I.S. Mudway, Oerle R. van, H.M. Spronk, F.R. Cassee, F.J. Kelly, R.M. Harrison, B. Brunekreef, E. Lebret, and N.A. Janssen. 2013. 'Components of ambient air pollution affect thrombin generation in healthy humans: the RAPTES project', *Occup Environ Med*, 70: 332-40.

Strak, M., N. Janssen, R. Beelen, O. Schmitz, I. Vaartjes, D. Karssenberg, C. van den Brink, M. L. Bots, M. Dijst, B. Brunekreef, and G. Hoek. 2017. 'Long-term exposure to particulate matter, NO2 and the oxidative potential of particulates and diabetes prevalence in a large national health survey', *Environ Int*, 108: 228-36.

Strak, M., N.A. Janssen, K.J. Godri, I. Gosens, I.S. Mudway, F.R. Cassee, E. Lebret, F.J. Kelly, R.M. Harrison, B. Brunekreef, M. Steenhof, and G. Hoek. 2012. 'Respiratory health effects of airborne particulate matter: the role of particle size, composition, and oxidative potential-the RAPTES project', *Environ Health Perspect*, 120: 1183-89.

Tonne, C., J.D. Yanosky, S. Beevers, P. Wilkinson, and F.J. Kelly. 2012. 'PM mass concentration and PM oxidative potential in relation to carotid intima-media thickness', *Epidemiology*, 23: 486-94.

Van Den Heuvel, R., E. Den Hond, E. Govarts, A. Colles, G. Koppen, J. Staelens, M. Mampaey, N. Janssen, and G. Schoeters. 2016. 'Identification of PM10 characteristics involved in cellular responses in human bronchial epithelial cells (Beas-2B)', *Environ Res*, 149: 48-56.

Van Den Heuvel, R., J. Staelens, G. Koppen, and G. Schoeters. 2018. 'Toxicity of Urban PM10 and Relation with Tracers of Biomass Burning', *Int J Environ Res Public Health*, 15.

vanMaanen, J.M.S., P.J.A. Borm, A. Knaapen, M. vanHerwijnen, P.A.E.L. Schilderman, K.R. Smith, A.E. Aust, M. Tomatis, and B. Fubini. 1999. 'In vitro effects of coal fly ashes: Hydroxyl radical generation, iron release, and DNA damage and toxicity in rat lung epithelial cells', *Inhal Toxicol*, 11: 1123-41.

Velali, E., E. Papachristou, A. Pantazaki, T. Choli-Papadopoulou, S. Planou, A. Kouras, E. Manoli, A. Besis, D. Voutsa, and C. Samara. 2015. 'Redox activity and in vitro bioactivity of the water-soluble fraction of urban particulate matter in relation to particle size and chemical composition', *Environ. Pollut*.

Verma, V., A. Polidori, J.J. Schauer, M.M. Shafer, F.R. Cassee, and C. Sioutas. 2009. 'Physicochemical and toxicological profiles of particulate matter in Los Angeles during the October 2007 southern California wildfires', *Environ. Sci. Technol*, 43: 954-60.

Weichenthal, S. A., E. Lavigne, G. J. Evans, K. J. Godri Pollitt, and R. T. Burnett. 2016. 'Fine Particulate Matter and Emergency Room Visits for Respiratory Illness. Effect Modification by Oxidative Potential', *Am J Respir Crit Care Med*, 194: 577-86.

Weichenthal, S., D. L. Crouse, L. Pinault, K. Godri-Pollitt, E. Lavigne, G. Evans, A. van Donkelaar, R. V. Martin, and R. T. Burnett. 2016. 'Oxidative burden of fine particulate air pollution and risk of cause-specific mortality in the Canadian Census Health and Environment Cohort (CanCHEC)', *Environ Res*, 146: 92-9.

Weichenthal, S., E. Lavigne, G. Evans, K. Pollitt, and R. T. Burnett. 2016. 'Ambient PM2.5 and risk of emergency room visits for myocardial infarction: impact of regional PM2.5 oxidative potential: a case-crossover study', *Environ Health*, 15: 46.

Wessels, A., W. Birmili, C. Albrecht, B. Hellack, E. Jermann, G. Wick, R.M. Harrison, and R.P. Schins. 2010. 'Oxidant generation and toxicity of size-fractionated ambient particles in human lung epithelial cells', *Environ. Sci. Technol*, 44: 3539-45.

Yang, A., N. A. Janssen, B. Brunekreef, F. R. Cassee, G. Hoek, and U. Gehring. 2016. 'Children's respiratory health and oxidative potential of PM2.5: the PIAMA birth cohort study', *Occup Environ Med*, 73: 154-60.

Zhang, X., N. Staimer, T. Tjoa, D. L. Gillen, J. J. Schauer, M. M. Shafer, S. Hasheminassab, P. Pakbin, J. Longhurst, C. Sioutas, and R. J. Delfino. 2016. 'Associations between microvascular function and short-term exposure to traffic-related air pollution and particulate matter oxidative potential', *Environ Health*, 15: 81.
